# Tuning the Selectivity of Catalytic Nitrile Hydrogenation with Phase-Controlled Co Nanoparticles Prepared by Hydrosilane-Assisted Method

**DOI:** 10.1021/jacs.4c04780

**Published:** 2024-07-18

**Authors:** He Jiang, Dian Deng, Yusuke Kita, Masashi Hattori, Keigo Kamata, Michikazu Hara

**Affiliations:** † Laboratory for Materials and Structures, Tokyo Institute of Technology, 4259 Nagatsuta, Midori-ku, Yokohama 226-8503, Japan; ‡ Department of Chemistry and Bioengineering, Graduate School of Engineering, Osaka Metropolitan University, 3-3-138 Sugimoto, Sumiyoshi-ku, Osaka 558-8585, Japan

## Abstract

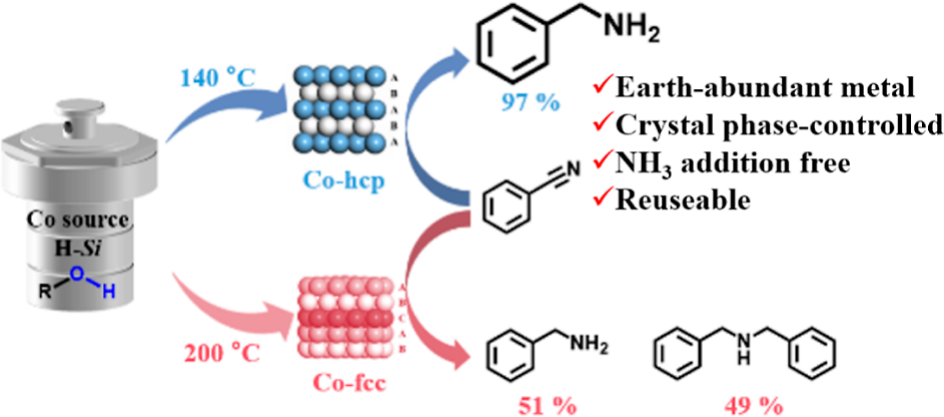

Cobalt (Co) is a promising candidate to replace noble metals in the hydrogenation process, which is widely employed in the chemical industry. Although the catalytic performance for this reaction has been considered to be significantly dependent on the Co crystal phase, no satisfactory systematic studies have been conducted, because it is difficult to synthesize metal nanoparticles that have different crystalline structures with similar sizes. Here we report a new method for the synthesis of cobalt nanoparticles using hydrosilane as a reducing agent (hydrosilane-assisted method). This new method uses 1,3-butanediol and propylene glycol to successfully prepare fcc and hcp cobalt nanoparticles, respectively. These two types of Co nanoparticles have similar sizes and surface areas. The hcp Co nanoparticles exhibit higher catalytic performance than fcc nanoparticles for the hydrogenation of benzonitrile under mild conditions. The present hcp Co catalyst is also effective for highly selective benzyl amine production from benzonitrile without ammonia addition, whereas many catalytic systems require ammonia addition for selective benzyl amine production. Mechanistic studies revealed that the fast formation of the primary amine and the prevention of condensation and secondary amine hydrogenation promote selective benzonitrile hydrogenation for benzylamine over hcp Co nanoparticles.

## 1 Introduction

Crystal phase engineering of nanomaterials stands as a crucial aspect of materials science that presents versatile applications in various fields such as semiconductors,^1^ magnetic materials,^2^ energy storage,^3^ optical and photonic materials,^4^ and catalysis.^5−20^ The application of crystal phase engineering has held particular significance in recent years, notably in catalysis, where it has garnered substantial attention for enhancement of the performance of nanoparticle (NP) catalysts. A number of investigations have shown that crystal-phase engineering can be a promising approach to modulate the performance of catalysts by alteration of the electronic and geometric structures of catalyst surfaces.^6^ Cobalt catalysts are increasingly regarded as promising alternatives to noble metallic catalysts due to their efficiency, environmental friendliness, and high abundance on Earth.^21^ These catalysts have gained prominence, particularly in the Fischer–Tropsch synthesis within industrial applications, due to their commendable intrinsic activity at low temperatures.^22^ Cobalt typically exists in bulk form under ambient conditions, primarily as either the hcp or fcc phase. It is noteworthy that while cobalt NPs exhibit stability in the fcc phase, the hcp phase transforms into the fcc phase at elevated temperatures, typically around 450 °C.^23^ The preparation of cobalt catalysts for catalytic applications often necessitates high-temperature treatments, such as wet impregnation methods,^24,25^ and high temperature accelerates the sintering of metallic Co; therefore, it is difficult to synthesize metallic Co hcp and fcc NPs with a similar particle size and surface area for discussion of the difference in catalytic activity of each crystal phase.^26−32^ Qin et al. devised a method to utilize an electric plasma discharge within an ultrasonic cavitation field of liquid ethanol to fabricate hcp and fcc Co NPs encapsulated in graphitic shells, whereby crystal phase control was achievable at annealing temperatures that exceeded 460 °C.^28^ In contrast, Guo et al. synthesized 200–230 nm hcp and fcc NPs using an ethanol/hydrazine/alkaline system at room temperature, but encountered difficulties in control of the crystal phase, which resulted in a mixed phase composition.^29^ These findings have underscored the ongoing challenge in the development of methodologies for the synthesis of Co NPs with controllable hcp or fcc structures at lower temperatures.

Here we have focused on the synthesis of transition metal NPs under mild conditions through the use of hydrosilanes as reducing agents; no additional reduction or capping solvents are employed, which results in the production of well-defined pure crystal NPs. We have previously reported that Ni NPs can be readily synthesized by the reduction of nickel complexes in the presence of hydrosilanes (hydrosilane-assisted method).^20^ Hydrosilanes were expected to act not only as reducing agents for Co cations, but also as ligands on the metal complexes to control the growth of metal particles; therefore, the synthesis of cobalt NPs with a controlled crystal phase was expected to be achieved by the addition of appropriate coordination compounds and a change in the structure of the diol solvents. The Co NPs with hcp structure exhibited much higher catalytic performance for selective productions of primary amines than those with fcc structures.

## 2 Experimental Section

### 2.1 Catalyst Preparation

#### 2.1.1 Preparation of hcp and fcc Structured Co NPs

Si–Co-fcc and Si–Co-hcp NPs were prepared by the hydrosilane-assisted method, in which hydrosilane acts as a reducing agent for the cobalt salt under solvothermal conditions. The selective preparation of Si–Co-hcp and Si–Co-fcc can be achieved by a simple change in the structure of the diol additive. For the typical synthesis of Si–Co-hcp NPs, 1 mmol of Co(OAc)_2_·4H_2_O was dissolved in 1 mL of propylene glycol (PG) and 4 mL of toluene in a glass tube, and the mixture was stirred at room temperature. After Co(OAc)_2_·4H_2_O was uniformly dispersed in the bottom solvent (PG), 5 mmol of phenylsilane was added to the mixed organic solution. The glass tube was then transferred into a 20 mL stainless steel autoclave, to which H_2_ at 2 MPa was injected. This was followed by a solvothermal process at 140 °C for 1 h under high-speed (550 rpm) stirring. After cooling to room temperature, the solution was filtered, washed several times with deionized water and acetone, and then dried in air for 2 h. The obtained solid was denoted as Si–Co-hcp NPs. The synthesis of Si–Co-fcc NPs was similar to that of Si–Co-hcp, except for an increase of the reaction temperature to 200 °C and replacement of the PG and toluene mixed solvent with 1,3-butanediol. The preparation of Si–Co-fcc was conducted in air without the addition of an H_2_ atmosphere. The samples synthesized under different reaction conditions are listed in Table 1.

**Table 1:** Reaction Conditions for Synthesis of fcc and hcp NPs^a^

entry	Co source	amount of phenylsilane (mmol)	solvent (5 mL)	temp. (°C)	crystal phase
1^b^	Co(OAc)_2_·4H_2_O	5	PG + Toluene	140	hcp (Si–Co-hcp)
2^b^	Co(OAc)_2_·4H_2_O	3	1,3-butanediol	200	fcc (Si–Co-fcc)
3	Co(OAc)_2_	3	1,3-butanediol	200	fcc
4	Co(OH)_2_	3	1,3-butanediol	200	hcp
5^c^	Co(acac)_3_	3	1,3-butanediol	200	hcp
6^d^	Co(acac)_2_	3	1,3-butanediol	200	hcp
7^d^^e^	CoCl_2_	3	1,3-butanediol	200	hcp
8^e^	Co(NO_3_)_2_·6H_2_O	3	1,3-butanediol	200	none

a All synthesis were run for 1 h.

b Typical synthesis of Co NPs with hcp and fcc structure.

c Foil was generated.

d Crystal intensity is pretty low.

e Incomplete reduction.

#### 2.1.2 Base Treatment to Remove Silicon (Si) Species from Co NPs and H_2_ Reduction of Co NP Surfaces

Si species are bonded on the surfaces of synthesized Si–Co-hcp and fcc NPs, which can prevent the catalytic activity of the metallic Co surface. Therefore, the surface Si species were removed from the Co NPs by treatment with a base. The base treatment process involved the addition of 200 mg of Co NPs to 50 mL of a 2 M NaOH methanol solution and stirring at 500 rpm for 5 h at room temperature in air. The solution was then filtered, and the treated cobalt NPs were washed several times with deionized water and methanol, and then dried in air for 2 h. The obtained solid was denoted as Co-hcp/fcc. The surfaces of the Si–Co-hcp/fcc and Co-hcp/fcc NPs were covered with Co oxide species, although the bulk in the particles is metallic Co (see below). The resultant Co-hcp/fcc NPs were reduced in a flow of H_2_ (flow rate = 30 mL min^–1^) at 200 °C for 2 h. The NPs were then transferred to an argon (Ar)-filled glovebox without exposure to the air. The reduced NPs were denoted as Reduced-Co-hcp/fcc.

#### 2.1.3 Preparation of HCP-Co NPs through β-Co(OH)_2_ Reduction^33^

The hexagonal β-Co(OH)_2_ was prepared using a simple precipitation method.^34^ A total of 3.0 g Co(NO_3_)_2_·6H_2_O was dissolved in 50 mL water to achieve a homogeneous solution using a magnetic stirrer. A total of 2.0 mol/L NaOH was added to the Co(NO_3_)_2_ solution dropwise. The mixture was filtered and washed with distilled water, and the formed Co(OH)_2_ was collected and dried at 60 °C under a vacuum overnight.

The two-dimensional HCP-Co was synthesized as follows: β-Co(OH)_2_ was placed in a quartz tube and heated to 320 °C at a rate of 10 °C/min under 10% Ar/H_2_ gas flow (1 bar, 60 sccm). After keeping at 320 °C for 2 h, the sample was passivated using a 1% O_2_/N_2_ mixture gas flow (1 bar, 40 sccm) for 3 h at room temperature. Then, the HCP-Co NPs was obtained.

### 2.2 Catalyst Evaluation

All experiments were performed under an anhydrous Ar atmosphere unless otherwise stated. The hydrogenation reactions were conducted in a 15 mL glass tube equipped with a magnetic stirrer. Substrates of 5 mL of toluene and 20 mg of catalyst were loaded into the glass tube reactor, which was then transferred into a 25 mL stainless steel autoclave. This procedure was performed in the Ar-filled glovebox. The stainless steel autoclave was subsequently moved to atmospheric conditions and H_2_ was injected into it. The autoclave was then placed into a preheated heater.

### 2.3 Characterization

Powder X-ray diffraction (XRD; Ultima IV, Rigaku) patterns were obtained using Cu Kα radiation (1.5405 Å, 40 kV, 40 mA) in the 2θ range of 20–90°. Nitrogen adsorption–desorption isotherms were measured at −196 °C with a surface-area analyzer (BELSORP-mini II, MicrotracBEL) to estimate the Brunauer–Emmett–Teller (BET) surface areas. X-ray photoelectron spectroscopy (XPS; ESCA-3200, Shimadzu, Mg Kα, 8 kV, 30 mA) was performed using the method as ref (25). The binding energies were calibrated using the C 1s peak (284.6 eV). Scanning transmission electron microscopy (STEM) measurements were conducted using a transmission electron microscopy system (JEM-ARM200F, JEOL, Japan) operated at 200 kV to provide beam spot widths of 0.2 nm, and energy-dispersive X-ray spectroscopy (EDS) measurements were performed with a 100 mm^2^ silicon drift detector (JED-2300, JEOL). Temperature-programmed desorption (TPD) and H_2_-pulse chemisorption were conducted using a chemisorption analyzer (BELCAT-A, BEL Japan) equipped with a thermal conductivity detector. CO_2_-TPD, NH_3_-TPD, and Substrate-TPD measurements were performed following the methodology outlined in ref (25). For Substrate-TPD, samples were adsorbed with substrates (2 mL of substrates for 180 mg of samples) for 3 h and subsequently dried and evacuated in a glovebox. The pretreatment for Substrate-TPD involved an Ar flow (30 sccm) for 1 h. In H_2_-pulse chemisorption, the catalyst was first reduced at 200 °C for 1 h under a pure H_2_ flow and then cooled to room temperature under an Ar atmosphere. H_2_ pulses (∼0.6498 cm^3^ per pulse) were injected until the eluted peak area of consecutive pulses remained constant. Inductively coupled plasma atomic emission spectroscopy (ICP-AES; ICPS-8100, Shimadzu) was performed to measure the amounts of leached metals. The sample preparation followed the method outlined previously.^24^ Gas chromatography (GC; GC-2025A, Shimadzu) analyses and mass spectrometry measurements were conducted using the same equipment and parameters as detailed in ref (24). Isolation of the product was performed with a single-channel automated flash chromatography system (Smart Flash EPCLC AI-580S, Yamazen). Nuclear magnetic resonance (NMR) spectra were recorded on Bruker Avance III-400 spectrometers (^1^H, 400 MHz; ^13^C, 100 MHz). All ^1^H NMR chemical shifts were recorded in ppm (δ) relative to tetramethyl silane or referenced to the chemical shifts of residual solvent resonances (CHCl_3_ was used as an internal standard, δ 7.26). All ^13^C NMR chemical shifts were recorded in ppm (δ) relative to carbon resonances in CDCl_3_ at δ 77.16. The adsorption process was conducted in a 5 mL glass bottle equipped with a magnetic stirrer inside the glovebox. Five mg of benzylamine, 5 mg of *N*-benzylidenebenzylamine, 10 mg of chlorobenzene, 2 mL of toluene, and 200 mg of catalyst were loaded in the bottle. Chlorobenzene was employed as the internal standard for GC measurements. Fourier transform infrared (FT-IR) spectra were obtained following the procedure outlined in ref (20).

## 3 Results & Discussion

### 3.1 Synthesis and Characterization of Cobalt hcp and fcc NPs

**Figure 1:**
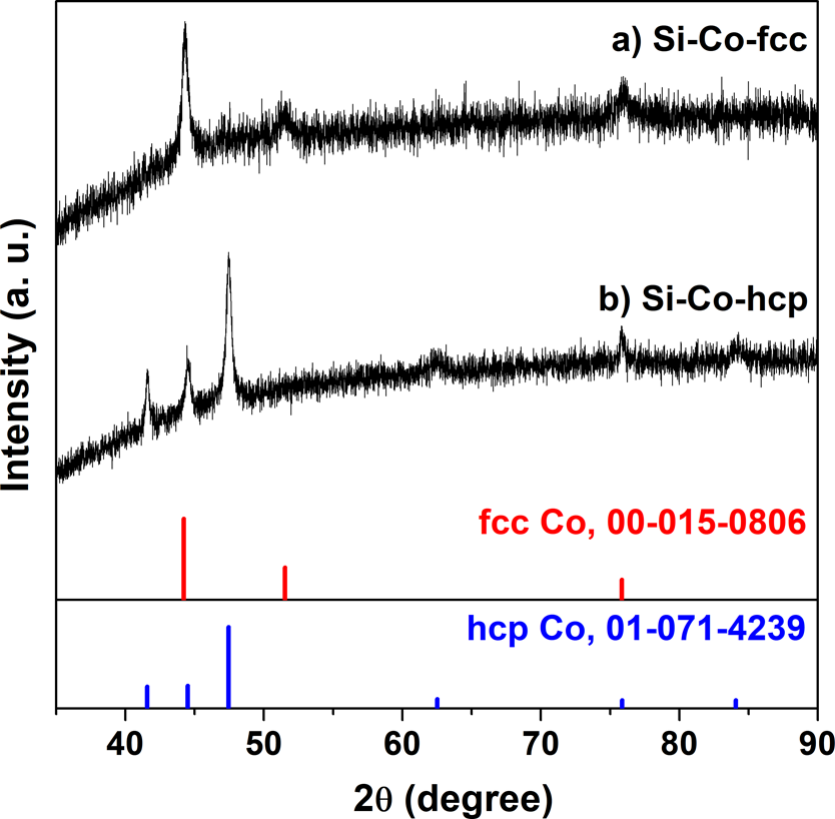
XRD patterns for (a) Si–Co-fcc and (b) Si–Co-hcp.

The Co NPs that featured both hcp and fcc crystal structures were typically synthesized through the reduction of the stable and economical cobalt(II) complex, Co(OAc)_2_·4H_2_O, with phenylsilane employed as a reducing agent. Distinct diols were strategically employed as solvents (PG for hcp and 1,3-butanediol for fcc structures) at various temperatures to modulate the crystalline architecture of the resultant Co NPs. Co(OAc)_2_·4H_2_O in a mixture of PG and toluene resulted in the hcp structure (Si–Co-hcp) (entry 1, Table 1), which was corroborated through XRD analysis (Figure 1a). The production of cobalt NPs with the fcc crystalline phase was also investigated thoroughly, which necessitated a high solvothermal temperature of 200 °C for reduction of the cobalt(II) complex into the fcc structure.^35^ The crystal phase intensity was significantly reduced at low temperatures in this system, which resulted in the synthesis of a mixture of Co NPs with hcp and fcc crystal phases (Figure S1). Solvents such as ethylene glycol, PG, 1,3-butanediol, toluene, 1,3,5-trimethylbenzene, dimethylformamide (DMF) and *N*,*N*′-dimethylpropyleneurea (entry 2 in Tables 1, S1, and Figure S2) were screened. 1,3-Butanediol yielded pure cobalt NPs with the fcc structure (Si–Co-fcc) as evidenced from XRD patterns (Figure 1b). To assess the impact of different cobalt sources on fcc Co NP preparation, 5 mL of 1,3-butanediol was employed as the solvent, along with various cobalt compounds such as Co(OH)_2_, Co(acac)_3_, Co(acac)_2_, CoCl_2_ and Co(NO_3_)_2_·6H_2_O, commonly used for Co NP synthesis (entries 2–8, Table 1).^36^ However, Co(OH)_2_, Co(acac)_3_, Co(acac)_2_, CoCl_2_ only yielded Si–Co-hcp NPs (see Figure S3). Furthermore, Co(NO_3_)_2_·6H_2_O was resistant to reduction under the same reaction conditions, so that no NPs were formed (see Figure S3). Consequently, only the protocol outlined in entry 2 offered an economical method for the production of pure phase fcc Co NPs. The physical parameters, such as the size and crystal phases, for transition metal NPs have been reported to be significantly influenced by the choice of metal salt and solvent, through modulation of the nucleation rate during synthesis.^17,27,35^ While the combination of 1,3-butanediol and Co(OAc)_2_·4H_2_O or Co(OAc)_2_ was effective for the preparation of fcc Co, there is no satisfactory explanation for the mechanism at present. The hcp Co and fcc Co NPs obtained were denoted as Si–Co-hcp and Si–Co-fcc, respectively.

**Figure 2:**
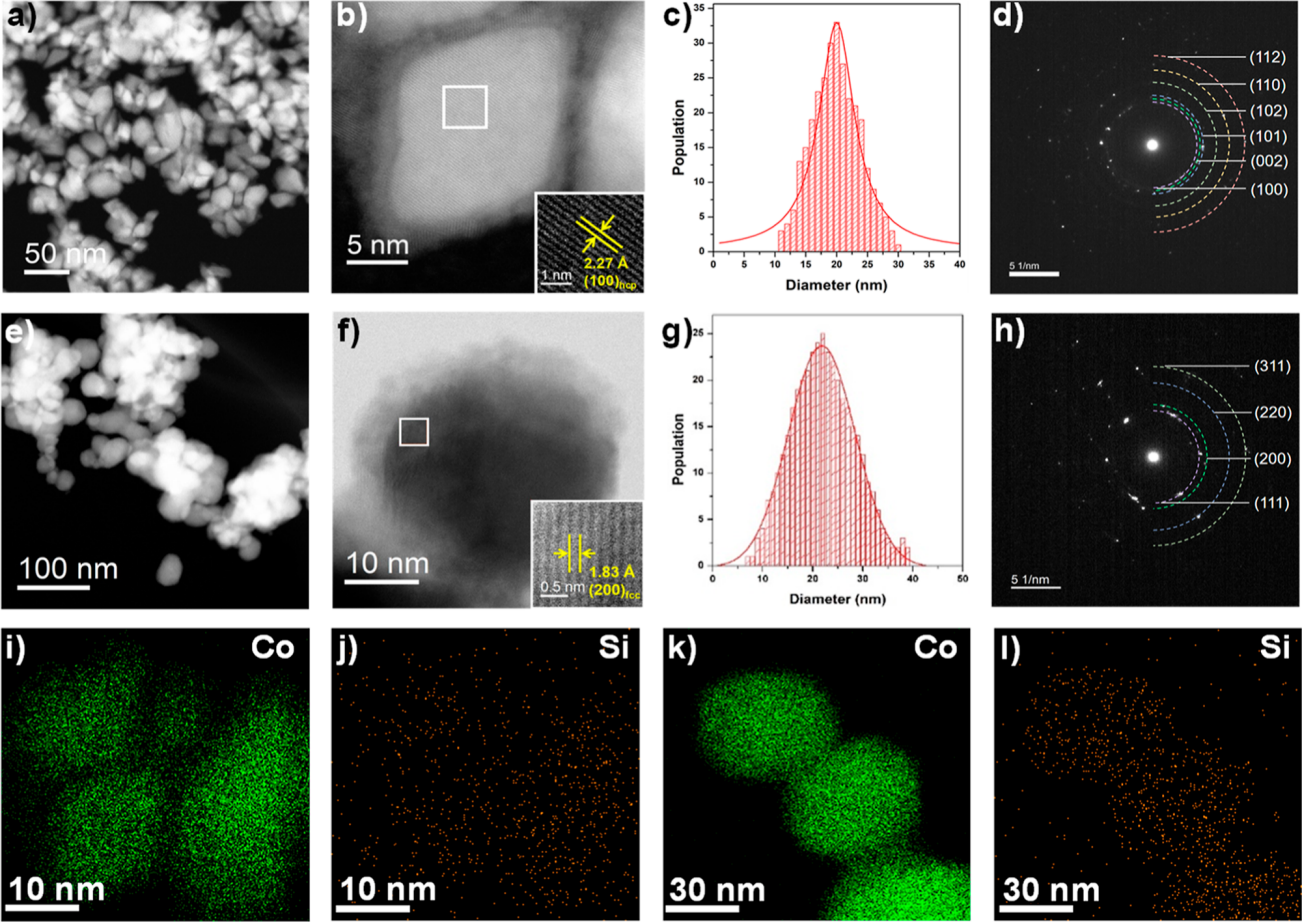
STEM images of (a,b) Si–Co-hcp, (e,f) Si–Co-fcc, diameter distributions of (c) Si–Co-hcp, (g) Si–Co-fcc, electron diffraction of (d) Si–Co-hcp, (h) Si–Co-fcc, STEM–EDX maps of Si–Co-hcp in (i) Co, (j) Si, and Si–Co-fcc in (k) Co, (l) Si.

Structural and elemental analyses were conducted using STEM imaging (Figure 2a,b,e,f) in conjunction with EDS elemental mapping (Figure 2i–l). The established synthesis procedure facilitated the formation of distinct cobalt NPs, which yielded 20 nm rhombic Si–Co-hcp NPs and 23 nm spherical Si–Co-fcc NPs separately (Figure 2c,g). The selected area electron diffraction patterns shown in Figure 2d,h revealed diffraction rings that corresponded to specific crystal planes, notably (112), (110), (102), (101), (002), and (100) for the Co hcp crystal, and (311), (220), (200), and (111) for the Co fcc crystal.^32^ These findings were consistent with the XRD patterns for Si–Co-hcp and -fcc (Figure 1). The EDS mapping of the Si–Co-hcp and Si–Co-fcc NPs verified the presence of cobalt and silicon as constituent elements with homogeneous distributions within the cobalt NPs (Figure 2i–l).

In the hydrosilane-assisted synthesis, both Si–Co-hcp and Si–Co-fcc NPs were covered by Si species, which originated from phenylsilane employed as the reductant for the conversion of Co(OAc)_2_·4H_2_O into metallic cobalt NPs. Comprehensive XPS analyses were performed in the Co 2p and Si 2s regions (Figure S4) to elucidate the specific composition of the Si species and the atomic concentrations of Co and Si. The peaks in the Si region of both the Si–Co-hcp and -fcc NPs resembled each other, with those at 153.4 eV signifying oxidized silicon.^37,38^ There was a large difference in the ratio of Si to Co between Si–Co-hcp and Si–Co-fcc, which was plausibly attributed to the higher synthesis temperature of Si–Co-fcc or the structure of the diol solvents (Table S2). The FT-IR spectrum for Si–Co-hcp indicated that the diols are incorporated as a silyl ether (Figure S5). The specific surface area of both the Si–Co-fcc and -hcp NPs was determined to be 6 m^2^ g^–1^, as ascertained through BET analysis (Figure S6a,b and Table S3). The hydrosilane-assisted synthesis method consequently yielded Si–Co-hcp and Si–Co-fcc NPs with consistent characteristics, which encompassed surface area and particle size; however, large differences were observed in the surface coverage by Si species.

We do not currently have any satisfactory explanation for the relationship between the formation mechanism single crystal Co NPs and the synthesis method in this study at low temperature ≤200 °C. This is because it has not been clarified what transforms Co hcp NPs into fcc NPs at a mere ca. 450 °C.^39^ Thermodynamically, Co fcc is stable above 600 °C as shown in the phase diagram, which suggests that kinetics relates to the phenomenon. Table 1 implies that Co starting materials, solvent and preparation temperature contribute to the synthesis of Co hcp and fcc NPs; the decomposition process of the starting materials may play an indispensable role in the formation of Co single crystal NPs. One possibility is that diols can act as capping agent to regulate the particle growth. Such kinetics is currently under investigation.

### 3.2 Base Treatment for Co NPs

It is difficult to discuss the difference in catalytic performance between the Si–Co-hcp and Si–Co-fcc crystalline phases because the surfaces of both were oxidized and there was a difference in the amount of Si between both samples. To remove Si species from the surfaces of both the Si–Co-hcp and Si–Co-fcc NPs, a base treatment was conducted that employed a sodium hydroxide (NaOH) methanol solution. The base treatment, which is well-established for Si and SiO_2_ removal,^40^ was carried out using both 5 and 2 M NaOH concentrations in methanol for the treatment of the Si–Co-hcp NPs. However, 5 M NaOH reacted excessively with the Co NPs, which resulted in the formation of Co(OH)_2_ during the base treatment, subsequent filtration, and drying stages (Figure S7). Therefore, base treatment with 2 M NaOH solution in methanol was used as the standard procedure. Si–Co-hcp and Si–Co-fcc after base treatment were denoted as Co-hcp and Co-fcc, respectively.

**Figure 3:**
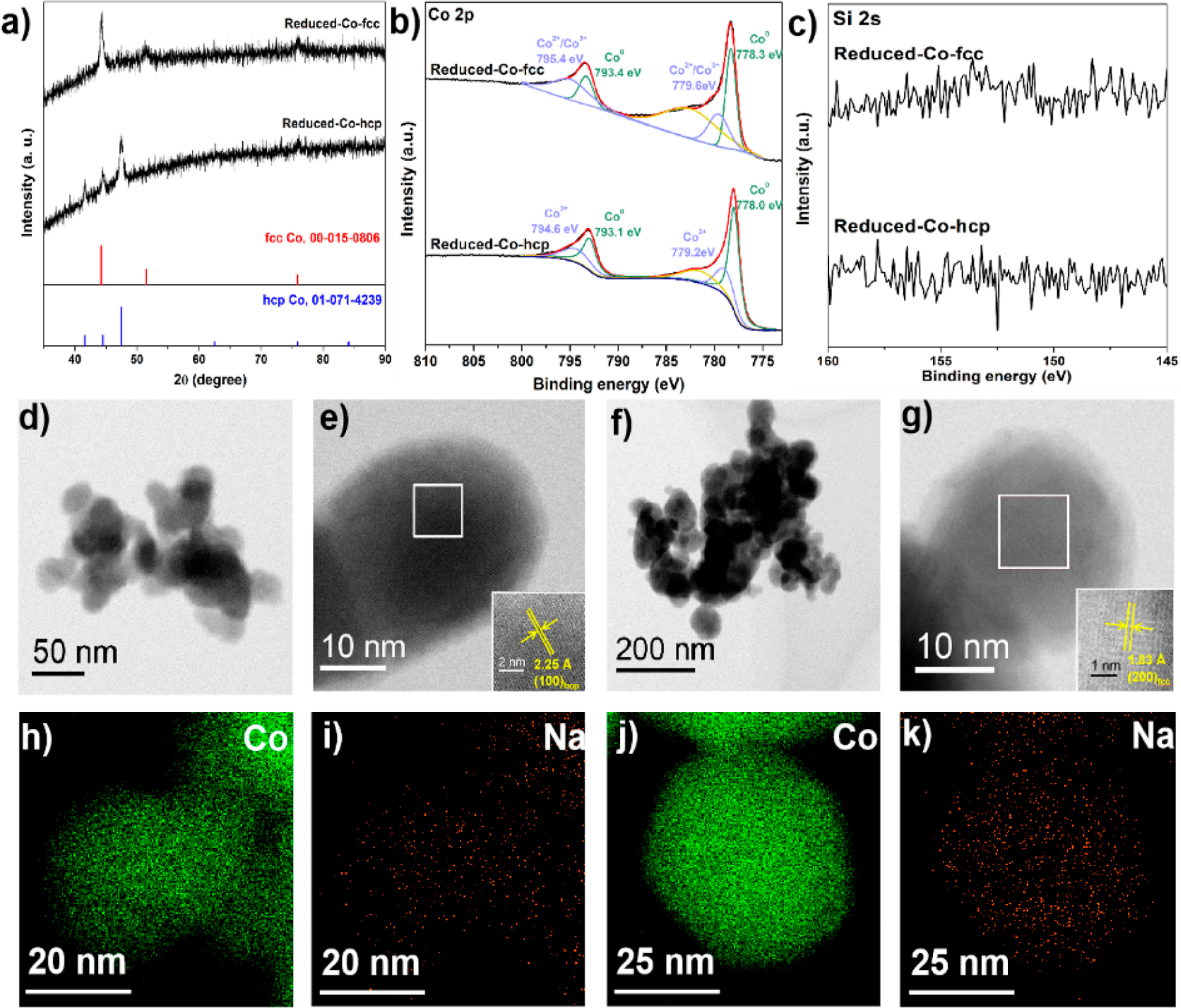
Characterization of typical Reduced-Co-hcp & Reduced-Co-fcc: (a) XRD patterns, XPS in (b) Co 2p region, (c) Si 2s region, STEM images of (d,e) Reduced-Co-hcp, (f,g) Reduced-Co-fcc, STEM–EDX maps of Reduced-Co-hcp in (h) Co, (i) Na, and Reduced-Co-fcc in (j) Co, (k) Na.

The Co NPs obtained after the base treatment and H_2_ reduction were characterized via XRD, XPS, TEM, and EDS elemental mapping (Figure 3). H_2_ reduction was required to reduce the surface Co(OH)_*x*_ species generated during the base treatment to metallic cobalt. The XRD analysis indicated there was no significant difference in the crystal phase after the base treatment (Figure 3a). Co 2p and Si 2s XPS spectra (Figure 3b,c) revealed that the base treatment, followed by H_2_ reduction resulted in metallic Co surfaces without Si species on Reduced-Co-hcp/fcc. STEM imaging also indicated a slight reduction in the diameter of the Co NPs, accompanied by a morphological transformation of the Si–Co-hcp NPs from rhombic to spherical (Figures 3d–g and S8). Moreover, EDS mapping of Reduced-Co-hcp and Co-fcc confirmed the presence of the constituent elements that included cobalt, sodium, and silicon (refer to Figures 3h–k and S9). The presence of sodium is ascribed to the strong affinity of Na species in NaOH. The detection of silicon indicated its presence not only on the surface but also within the bulk of the Co NPs (Table S4).

The specific surface area of both Reduced-Co-hcp and Co-fcc increased approximately 2-fold, from 6 to 11 m^2^ g^–1^ and 13 m^2^ g^–1^, respectively (Table S3 and Figure S6). It should be noted that the crystallite size calculated from XRD measurements using the Scherrer equation remained consistent with that for the untreated NPs at approximately 20 nm.^41^ Reduced-commercial Co-fcc NPs also had a similar specific surface area to that of Reduced-Co-hcp and Co-fcc NPs. H_2_ pulse chemisorption on Reduced-Co-hcp and Co-fcc revealed similar average H_2_ chemisorption capacities, with values of 0.157 cm^3^ g^–1^ for Reduced-Co-hcp and 0.198 cm^3^ g^–1^ for Reduced-Co-fcc. The slight difference observed may be attributed to the relatively larger specific surface area of Reduced-Co-fcc (13 m^2^ g^–1^) compared to Reduced-Co-hcp (11 m^2^ g^–1^). This indicates that the number of active sites for H_2_ chemisorption on Reduced-Co-hcp and Reduced-Co-fcc are comparable. Additional, CO_2_-TPD and NH_3_-TPD analyses show no acid and base sites on Reduced-Co-hcp and Reduced-Co-fcc (Figures S10 and S11). The comparison between cobalt NPs produced through our hydrosilane-assisted method and those detailed in reported literature highlights the distinctive capacity of our method for controlling crystal phases (Table S3).

### 3.3 Catalytic Performance

**Figure 4:**
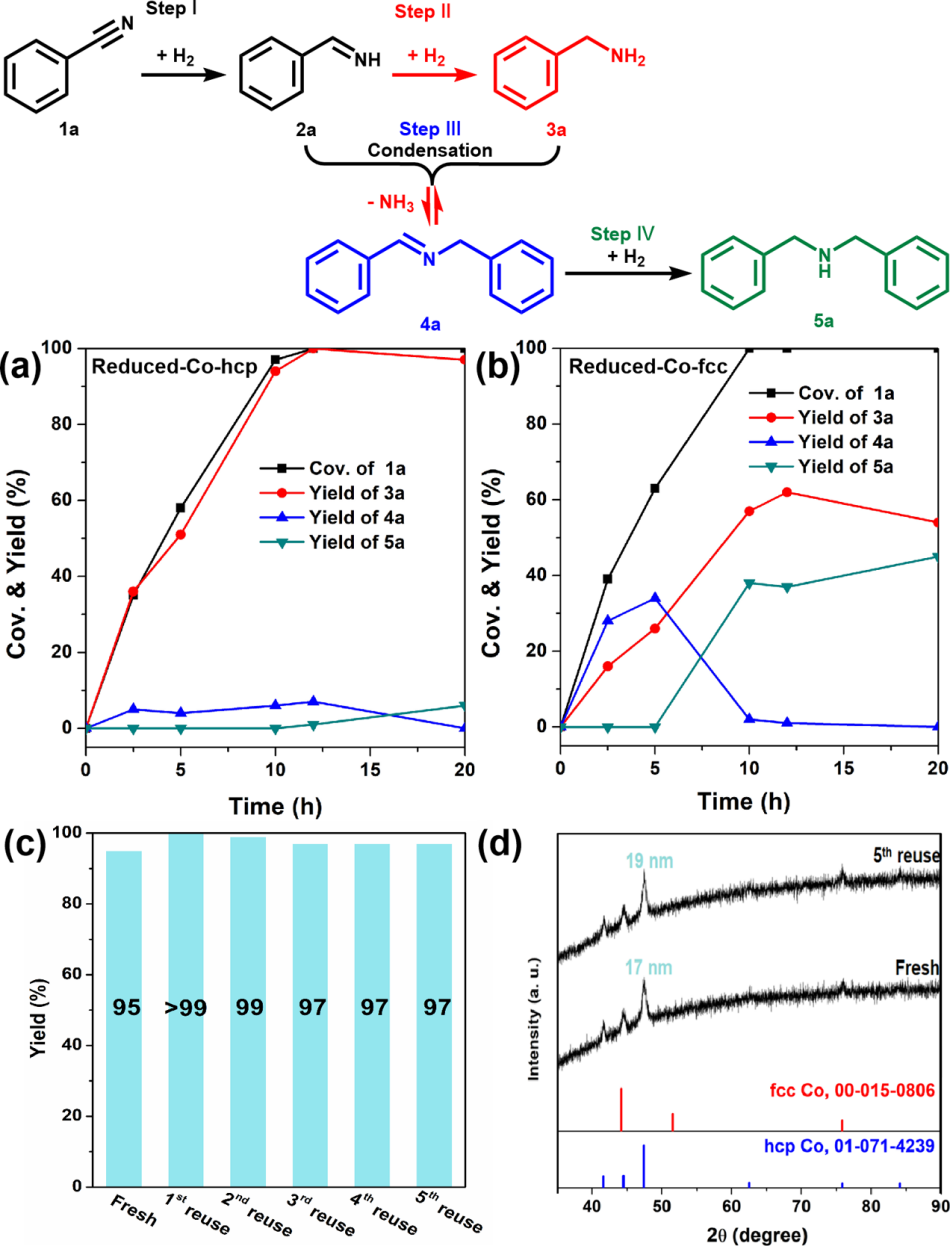
Possible reaction pathway of benzonitrile hydrogenation, and time course of benzonitrile hydrogenation over (a) Reduced-Co-hcp and (b) Reduced-Co-fcc. (c) Reuse experiment of benzonitrile hydrogenation over Reduced-Co-hcp. Reaction conditions: catalyst (20 mg), **1a** (0.5 mmol), toluene (5 mL), pH_2_ (0.5 MPa), 70 °C, 20 h. (d) XRD patterns of the fresh and fifth reused Reduced-Co-hcp.

The catalytic performance of the Reduced-Co-fcc and Co-hcp catalysts was investigated to assess the influence of the crystal phase on the hydrogenation of nitriles as a model reaction to yield primary amines, which are known as versatile intermediates and crucial precursors in the syntheses of a broad spectrum of compounds, including natural products, pharmaceuticals, dyes, pigments, agrochemicals, and polymers.^42^ During the hydrogenation process to convert nitriles (**1**) into primary amines (**2**), the formation of secondary amines (**4**) often occurs due to the hydrogenation of secondary imines (**3**), which are generated through the condensation of primary amine and primary imine intermediates, as schematically illustrated in Figure 4. Previously reported catalytic systems required ammonia to achieve high selectivity toward primary amines,^43−48^ except for four cobalt-based catalytic systems.^24,49−51^ Reduced-Co-hcp exhibited notably high activity and selectivity toward the hydrogenation of benzonitrile (**1a**) to give benzylamine (**3a**) in 97% yield, even without the addition of ammonia (entry 1, Table 2). On the other hand, Reduced-Co-fcc gave a mixture of **2a** and dibenzylamine (**5a**) under the same reaction conditions (entry 3). Although we have reported that Co-fcc/SiO_2_ shows high activity for nitrile hydrogenation without the addition of NH_3_,^24^ the selectivity toward the primary amine was lower than that of Reduced-Co-hcp (entry 4). The results for previously reported HCP-Co NPs are shown in entry 5 and 6. There was no difference in particle size and surface area between the HCP-Co NPs and Reduced-Co-hcp (Table S3). Nevertheless, the latter surpassed the former in catalytic activity. Purchased commercial Co fcc NPs produced by a high temperature process was also examined through the same reaction. Commercial Co fcc NPs were reduced by H_2_ as well as other Co NPs, and the impurities, including Si and Na, were not detected in the resulting Reduced-commercial Co-fcc by XPS and EDX. While Table 2 demonstrates that Reduced-commercial Co-fcc (entry 7) has a catalytic activity close to that of Reduced-Co-fcc, the conversion in both cases exceeds 99%; we cannot compare the kinetics of Reduced-Co-fcc with that of Reduced-commercial Co-fcc at such high conversion. For this reason, both catalysts were compared at low conversion (42–46%, Table S5). In Table S5, there was no significant difference in conversion and each product yield between Reduced-commercial Co-fcc and Reduced-Co-fcc. In the case of Reduced-Co-hcp and -fcc, Si species were not detected by XPS but EDX (Figure 3c and S9), which means that Si species remain in the bulk of Co NPs. On the other hand, from the synthesis procedure and the Na-EDX images in Figure 3i,k, Na species are expected to remain on the surface of Co NPs. Nevertheless, Reduced-commercial Co-fcc without impurities is similar to Reduced-Co-fcc, where Na and Si species remain in the surface and bulk of Co NPs, respectively, in catalytic performance. This suggests that these impurities have no significant effect on the catalysis of metallic Co surface. Reduced-Co-hcp showed higher selectivity for the hydrogenation of benzonitrile (**1a**) toward benzylamine (**3a**) than the reported Co catalysts under milder reaction conditions (Table S6).

**Table 2:**
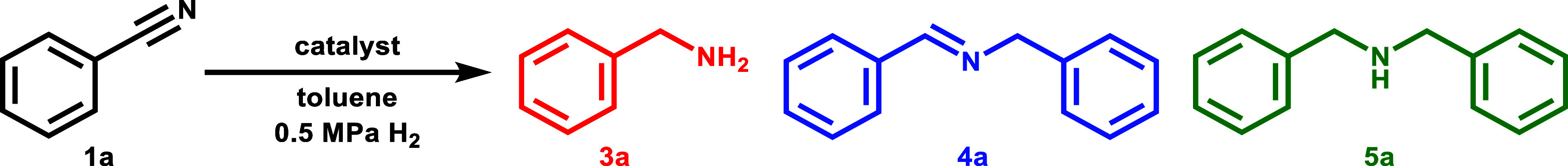
Hydrogenation of **1a** Over Co Catalysts^a^

					yield^b^ (%)		
entry	catalyst	temp. (°C)	time (h)	conv. (%)	**3a**	**4a**	**5a**
1	Reduced-Co-hcp	70	20	>99	97	-	3
2	Reduced-Co-hcp	70	5	58	51	4	-
3	Reduced-Co-fcc	70	20	>99	51	-̵	49
4^24^	Co-fcc/SiO_2_^c^	50	20	>99	78	3	19
5	HCP-Co NPs^d^	70	20	84	74	10	-
6	HCP-Co NPs^d^	70	5	30	23	6	-
7	Reduced-commercial Co-fcc	70	20	>99	69	-	30

a Reaction conditions: catalyst (20 mg), **1a** (0.5 mmol), toluene (5 mL), *p*H_2_ (0.5 MPa), 20 h.

b Determined by GC.

c Cyclohexane was used as a solvent.

d HCP-Co NPs is synthesized with the method in ref (33).

Time-dependent profiles of the conversion rates and product yields (**3a**, **4a**, and **5a**) during benzonitrile hydrogenation with the Reduced-Co-hcp and Co-fcc catalysts are presented in Figure 4a,b. Both catalysts exhibited almost identical conversion rates and achieved complete consumption of **1a** within 12 h. However, a pivotal distinction arose in the intermediate **4a**. Reduced-Co-hcp efficiently converted **1a** to **3a** with only marginal formation of **4a** during the reaction. In contrast, Reduced-Co-fcc resulted in the equimolar production of **3a** and **4a** within 5 h, which subsequently led to the formation of **5a** from **4a** in the latter stages of the reaction. The results of a catalyst reuse study with Reduced-Co-hcp at 70 °C are given in Figure 4c. These findings demonstrate the significant catalyst stability and sustained selectivity, even after five consecutive cycles, which attests to the robustness of Reduced-Co-hcp NPs throughout multiple usages. Leaching tests for Co via ICP-AES analysis of the reaction solution after the initial run, revealed negligible Co leaching (0.02%), which affirmed the structural integrity of the catalyst. XRD patterns for Reduced-Co-hcp NPs recovered after six cycles offered compelling evidence that the hcp crystal phase can be regarded as the active site for benzylamine synthesis because the crystal phase of the catalyst remained as hcp (Figure 4d). Furthermore, calculations based on the Scherrer equation suggest only a minor increase in the crystallite size of the nanocatalyst, from 17 to 19 nm after six cycles, which underscores the stability of the catalyst dimensions across multiple cycles under mild reaction conditions.

The substrate scope for the Reduced-Co-hcp catalyst was systematically investigated under optimized conditions (Table 3). Primary amines were selectively obtained, regardless of the electronic effects of substituents on the benzene ring of benzonitrile (entries 1–3). The substituent at the 2-position of benzonitrile had a small steric effect on the present reaction. Small methyl groups did not affect the reactivity of the substrate (entry 1 vs 4), whereas large methoxy groups retarded the reaction (entry 2 vs 5). The yield of 2-methoxybenzylamine remained unimproved, regardless of adjustments to the reaction temperature (Table S7). Nitriles bearing *N*-heterocycles exhibited significant reactivity with complete conversion achieved under mild conditions. However, the selectivity toward the corresponding primary amine was inferior to that of benzylamine, with a 71% yield of the primary amine and a 28% yield of the secondary amine (entry 7). This discrepancy may be attributed to the presence of basic pyridine rings in nitriles containing *N*-heterocycles, which promoted imine hydrogenation by coordination with *N*-containing organic compounds.^24^ In contrast to the aromatic nitriles, the catalytic hydrogenation of aliphatic nitriles presented notable challenges due to Reduced reactivity and the formation of byproducts that contained methyl moieties during the hydrogenation process.^52,53^ Nevertheless, this catalyst system demonstrated exceptional selectivity toward primary amines, not only for phenylacetonitrile, but also for aliphatic nitriles and dinitriles.

**Table 3:**
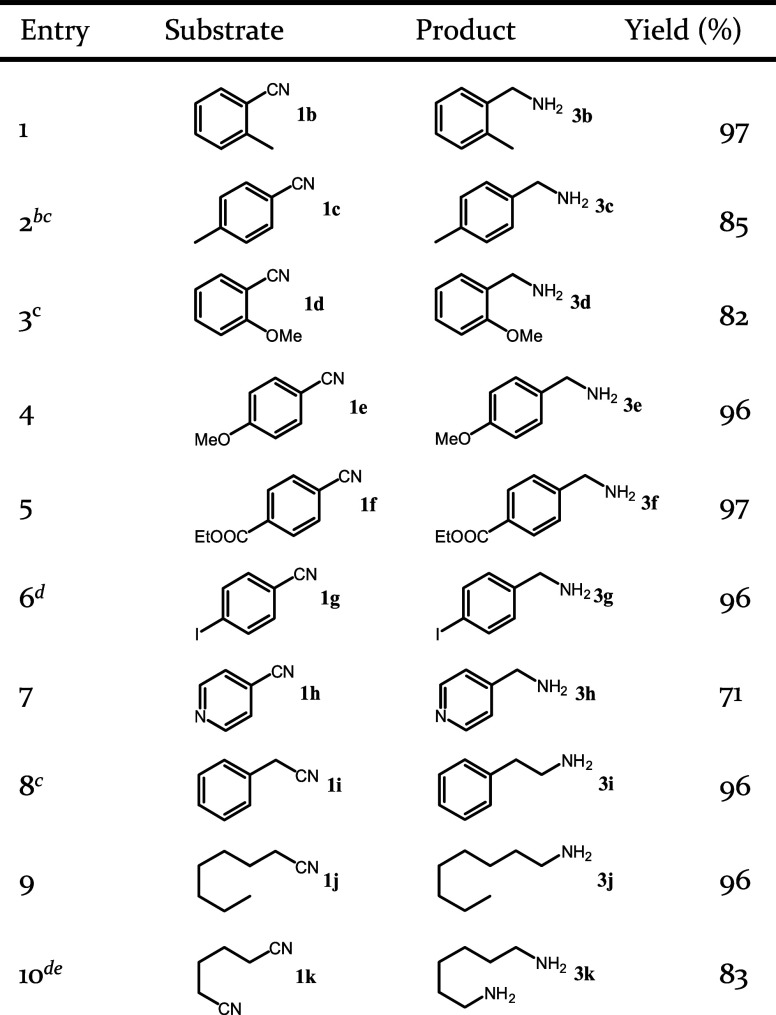
Hydrogenation of Nitriles over Reduced-Co-hcp^a^

a Reaction conditions: Reduced-Co-hcp (0.02 g), nitrile (0.5 mmol), toluene (5 mL), *p*H_2_ (0.5 MPa) at 70 °C, 20 h. Yields determined by ^1^H NMR spectroscopy with tetramethylsilane as an internal standard.

b Run at 60 °C for 40 h.

c Isolated yield.

d Run at 90 °C.

e Yields determined by GC.

### 3.4 Effects of Crystal Phase on Selectivity toward Nitrile Hydrogenation

Based on the compelling experimental results presented in Figure 3, Reduced-Co-hcp and Co-fcc gave completely different product distributions for nitrile hydrogenation, even though almost similar nitrile consumption rates were observed. In addition, catalyst characterization showed no significant differences in particle size, specific surface area. Therefore, the observed selectivity difference is considered to be due to the crystalline phase of cobalt. To elucidate the effects of the crystalline phase for selectivity toward nitrile hydrogenation, Reduced-Co-hcp and Co-fcc catalysts were compared at each reaction step during benzonitrile hydrogenation, as schematically illustrated in Figure 4. First, the hydrogenation of benzonitrile leads to the formation of the phenylmethanimine (**2a**) intermediate (step I). The subsequent hydrogenation of **2a** yields the desired primary amine **3a** (step II). A condensation reaction of **3a** with **2a** gives *N*-benzylidenebenzylamine (**4a**), accompanied by the release of ammonia (step III).^54^ The hydrogenation of **4a** forms dibenzylamine (**5a**) as a byproduct (step IV).

**Figure 5:**
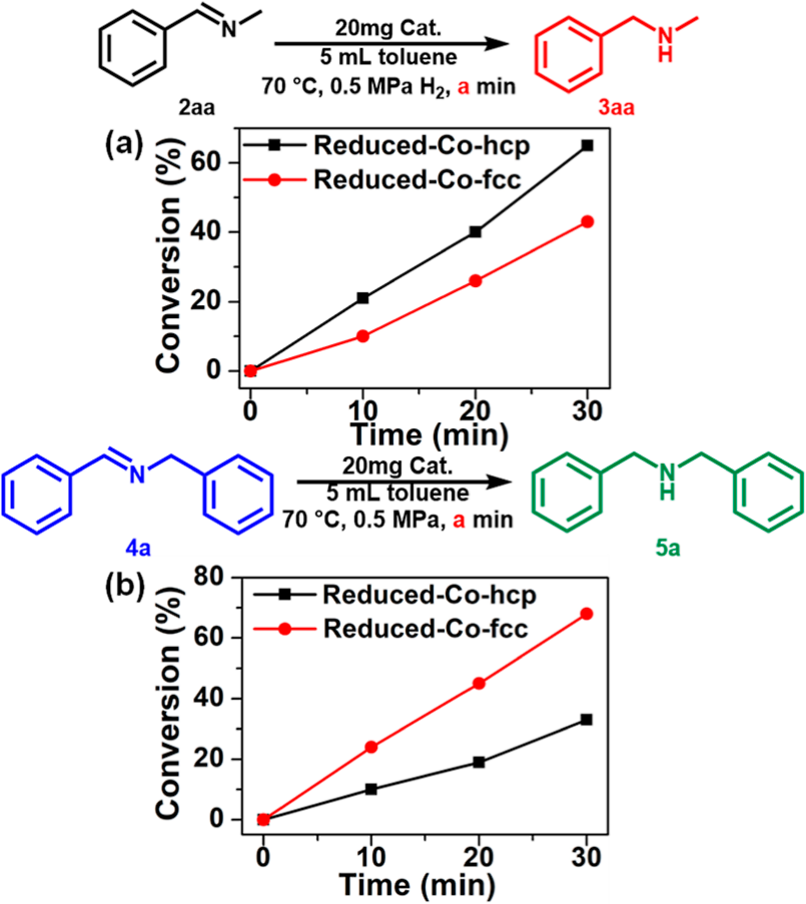
Catalytic performance on half-reactions on the Reduced-Co-hcp and Reduced-Co-fcc. Reaction conditions: substrates (0.5 mmol), catalyst (20 mg), toluene (5 mL), *p*H_2_ (0.5 MPa) at 70 °C. (a) *N*-Benzylidenemethanamine (**2aa**) hydrogenation. (b) *N*-benzylidenebenzylamine (**4a**) hydrogenation.

For step I, Reduced-Co-hcp and Co-fcc are considered to exhibit similar activities when the nitrile conversion rates over time are compared (Figures 4a vs 4b). Given the inherent instability of primary imine **2a** under ambient conditions, the comparison of activity for step II was performed using *N*-benzylidenemethanamine (**2aa**) as a suitable analog. The observed reaction rate with Reduced-Co-hcp surpassed that with Reduced-Co-fcc (Figure 5a). In addition, comparable experiments on the reductive amination of benzyldehyde aimed at simulation of the hydrogenation of primary imine were conducted under identical conditions using Reduced-Co-hcp and Co-fcc with 0.4 MPa NH_3_. The trend in the yield of the primary amine (Figure S12) was consistent with the hydrogenation of *N*-benzylidenemethanamine (Figure 5a). Regarding step III, the condensation of imines with amines has been reported to proceed smoothly in the absence of proton and metal catalysts.^55^ The condensation of *N*-methyl imine and **3a** reached equilibrium within 10 min at 70 °C without a catalyst. The same results were observed for the condensation of *N*-methyl imine and **3a** over Reduced Co-hcp or Co-fcc without H_2_ (Figure S13). These results indicate that the condensation step does not affect the selectivity toward nitrile hydrogenation over Co NPs. In step IV, Reduced-Co-fcc exhibited a higher conversion rate for the secondary imine compared to Reduced-Co-hcp (Figure 5b), in contrast to the observed hydrogenation activity in step II. Furthermore, the equimolar mixture of **2aa** and **4a** as substrates was subjected to hydrogenation under identical conditions using Reduced-Co-hcp and Co-fcc (Figure S14). The observed trend, consistent with the separate half-reactions, confirms the differential hydrogenation abilities of Reduced-Co-hcp and Co-fcc for primary imine and secondary imine. This discrepancy in hydrogenation capability may be attributed to the different adsorption capacities of Reduced-Co-hcp and Co-fcc for the primary amine and secondary imine,^24^ which was supported by the experimental findings. The competitive adsorption ratio for benzylamine and dibenzylimine over Reduced-Co-hcp and Co-fcc revealed substantial differences (Table S8). The adsorption of primary amine **3a** and secondary imine **4a** over Reduced-Co-hcp was marginal, which indicated its limited adsorption capacity for products and intermediates. Although the adsorption of primary imines cannot be confirmed by adsorption experiments due to their instability, the time course profile indicates that the continuous hydrogenation from nitriles to primary amines proceeds smoothly (Figure 4a). Therefore, the hydrogenation of primary imines over Reduced-Co-fcc proceeds without desorption from the catalyst surface. On the other hand, the formation of secondary imines is suppressed over Reduced-Co-hcp because the primary imine is readily hydrogenated on the catalyst surface to facilitate the desorption of the resulting primary amine, which results in the low concentration of secondary imines on the surface, as observed in the time course profile over Reduced-Co-hcp (Figure 4a); the high selectivity of Reduced-Co-hcp can be rationalized by the low concentration and weak adsorption of secondary imines. Conversely, for Reduced-Co-fcc, the adsorption ratios for **3a** and **4a** were 12 and 26%, respectively. The stronger adsorption of primary amines and secondary imines on Reduced-Co-fcc than that on Reduced-Co-hcp promotes the production of secondary imines on the catalyst surface and the hydrogenation of secondary imines, which leads to a decrease in selectivity. It should be noted that there was no significant difference in the desorption of **3a** and **4a** from Reduced-Co-hcp and fcc in the case of TPD experiment (Figure S15) where **3a** or **4a** was adsorbed on both surfaces in the absence of toluene as a solvent; the results are not consistent with those in Table S8 obtained by the adsorption of **3a** and **4a** in toluene. This suggests that the solvent contribute to and complicates the adsorption of **3a** and **4a** on Co hcp and fcc.

**Figure 6:**
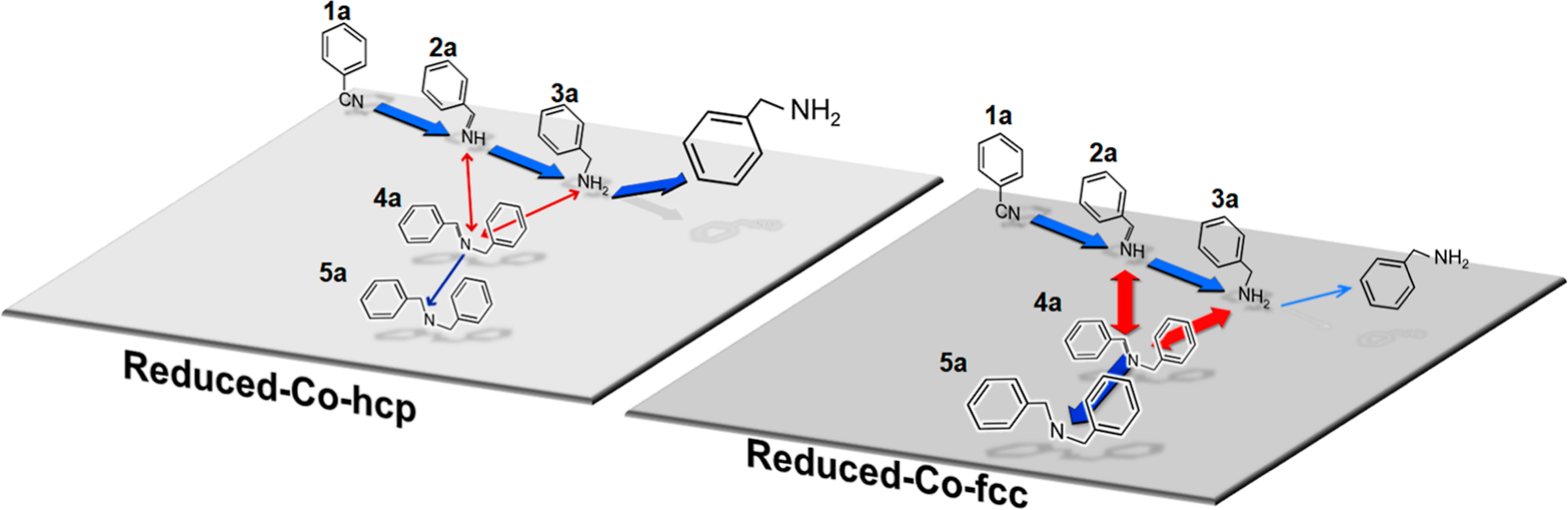
Proposed mechanism for the hydrogenation of **1a** over Reduced-Co-hcp and Reduced-Co-fcc.

On the basis of these results, we propose a possible mechanism for the hydrogenation of **1a** over Reduced-Co-hcp and Co-fcc (Figure 6). The hydrogenation of **1a** over Reduced-Co-hcp proceeded without desorption in the first step to give primary amine **3a**, which was easily desorbed from the catalyst surface. The adsorption of **3a** is suppressed on Reduced-Co-hcp; therefore, the formation of **4a** through condensation with **2a** is considerably slow, so that the catalyst exhibits high catalytic performance for primary amine formation. On the other hand, the condensation reaction between **3a** and **2a** is promoted because **3a** is easily adsorbed on Reduced-Co-fcc, which causes a high concentration of secondary imine **4a** in the reaction mixture. Such a difference in adsorption capability between Co hcp and fcc is also observed in hydrogen adsorption. H_2_ desorption peak on Co fcc (100) surface is observed at 260 K. On the other hand, H_2_ desorption peak on Co hcp (0001) surface appears at 350 K.^56^ This may be attributed to strongly spin-polarized Co hcp; the polarized d-bond of Co 3d orbitals crosses the Fermi level.^57^ The splitting of spin-polarized orbits causes the alleviation of the repulsive energy of more empty bands, which indicates that the subtle atomic arrangement difference between the Co hcp and Co fcc results in a quite obvious change in the local electronic environment on the Co 3d orbitals.

To summarize the above mechanistic studies, the heightened selectivity of benzylamine with Reduced-Co-hcp during benzonitrile hydrogenation stems from its limited adsorption capacity for benzylamine and dibenzylimine, as evidenced by the mechanism and competitive adsorption investigations. Although the distinctive adsorption capacities of Co NPs with various crystal phases are potentially attributed to their differing abilities across distinct facets,^7,13,56,58^ further investigations are required to discuss the selectivity difference due to the crystal phase.

## 4 Conclusions

We have presented a phase-controllable, one-pot synthesis method for the production of cobalt NPs with either a hcp or fcc crystal phase and a similar particle size and surface area using a combination of the readily available cobalt complex Co(OAc)_2_·4H_2_O and hydrosilane. NaOH-treated Co-hcp NPs exhibit superior selectivity for primary amine and reusability under low H_2_ pressurization and at low temperatures. Furthermore, this Co catalytic system displays compatibility with a diverse range of nitriles and carbonyl compounds. The exceptional selectivity toward benzylamine over Reduced-Co-hcp can be attributed to the efficient primary imine hydrogenation and the rate-limited condensation between primary imine and primary amine, along with the secondary imine hydrogenation process. Moreover, we propose a hypothesis that differences in the reaction kinetics may be due to the limited adsorption capacity of metallic Co-hcp surfaces for benzylamine and dibenzylimine. These properties can be attributed to a new type of NP synthesis method that selectively forms Co hcp or fcc NPs with a similar particle size and surface area.

## Supporting Information Available

Additional discussion, NMR spectroscopy (PDF)
