# Supplementary material for: Tuning the Selectivity of Catalytic Nitrile Hydrogenation with Phase-Controlled Co Nanoparticles Prepared by Hydrosilane-Assisted Method
Source: J Am Chem Soc. 2024 Jul 18;146(30):20919–29. doi: 10.1021/jacs.4c04780 (PMC11295180; doi:10.1021/jacs.4c04780)
Supplement: Supplementary file 1 — ja4c04780_si_001.pdf [file ja4c04780_si_001.pdf]

Supporting information for

Tuning the Selectivity of Catalytic Nitrile Hydrogenation with Phase-Controlled Co Nanoparticles Prepared by Hydrosilane-Assisted Method

He Jiang[a], Dian Deng[a], Yusuke Kita[b], Masashi Hattori[a], Keigo Kamata[a], Michikazu Hara[a]*

[a] Laboratory for Materials and Structures, Tokyo Institute of Technology, 4259 Nagatsuta, Midori-ku, Yokohama 226–8503, Japan

[b] Department of Chemistry and Bioengineering, Graduate School of Engineering, Osaka Metropolitan University, 3-3-138 Sugimoto, Sumiyoshi-ku, Osaka 558-8585, Japan

*E-mail: mhara@msl.titech.ac.jp

Contents

1. Materials and reagents
2. Supplementary Figures and Tables
3. NMR results
4. References

1. Materials and reagents

$Co(OAc)_2 \cdot 4H_2O$ (Fuji Film Wako Pure Chemical Industries), $Co(OAc)_2$ (thermo scientific), $Co(OH)_2$ (Sigma-Aldrich), $Co(acac)_2$ (Sigma-Aldrich), $Co(acac)_3$ (Sigma-Aldrich), $CoCl_2$ (Sigma-Aldrich), $Co(NO_3)_2 \cdot 6H_2O$ (Kanto Chemical), propylene glycol (Fuji Film Wako Pure Chemical Industries), toluene (Kanto Chemical), 1,3-butanediol (Tokyo Chemical Industries), ethylene glycol (Tokyo Chemical Industries), 1,3,5-trimethylbenzene (Tokyo Chemical Industries), dimethylformamide (DMF) (Kanto Chemical), 1,3-Dimethyl-3,4,5,6-tetrahydro-2(1H)-pyrimidinone (DMPU) (Tokyo Chemical Industries), phenylsilane (Sigma-Aldrich), acetone (Kanto Chemical), were used for the catalyst preparation.

NaOH (Kanto Chemical), Tetra-n-butylammonium fluoride (TBAF) (Kanto Chemical), methanol (Kanto Chemical) and tetrahydrofuran (THF) (Kanto Chemical) were used for the surface treatment.

The following solvents were used for catalytic reactions and characterizations during the research. Benzonitrile, *o*-tolunitrile, *p*-tolunitrile, 2-methoxybenzonitrile, anisonitrile, ethyl 4-cyanobenzoate, phenylacetonitrile, 4-iodobenzonitrile, octanenitrile, adiponitrile, 4-cyanopyridine, 4-picolylamine, 1,6-diaminohexane, were supplied by Tokyo Chemical Industries. Chloroform-D, were supplied by Kanto Chemical. N-benzylidenebenzylamine, were supplied by Fuji Film Wako Pure Chemical Industries. Benzylamine, dibenzylamine, *N*-benzylidenemethanamine, *N*-benzylmethylamine, benzyl alcohol, were supplied by Sigma-Aldrich.

Cobalt powder (commercial-Co-fcc) was purchased from io-li-tec nanomaterials for comparing the catalytic performance.

2. Supplementary tables and figures

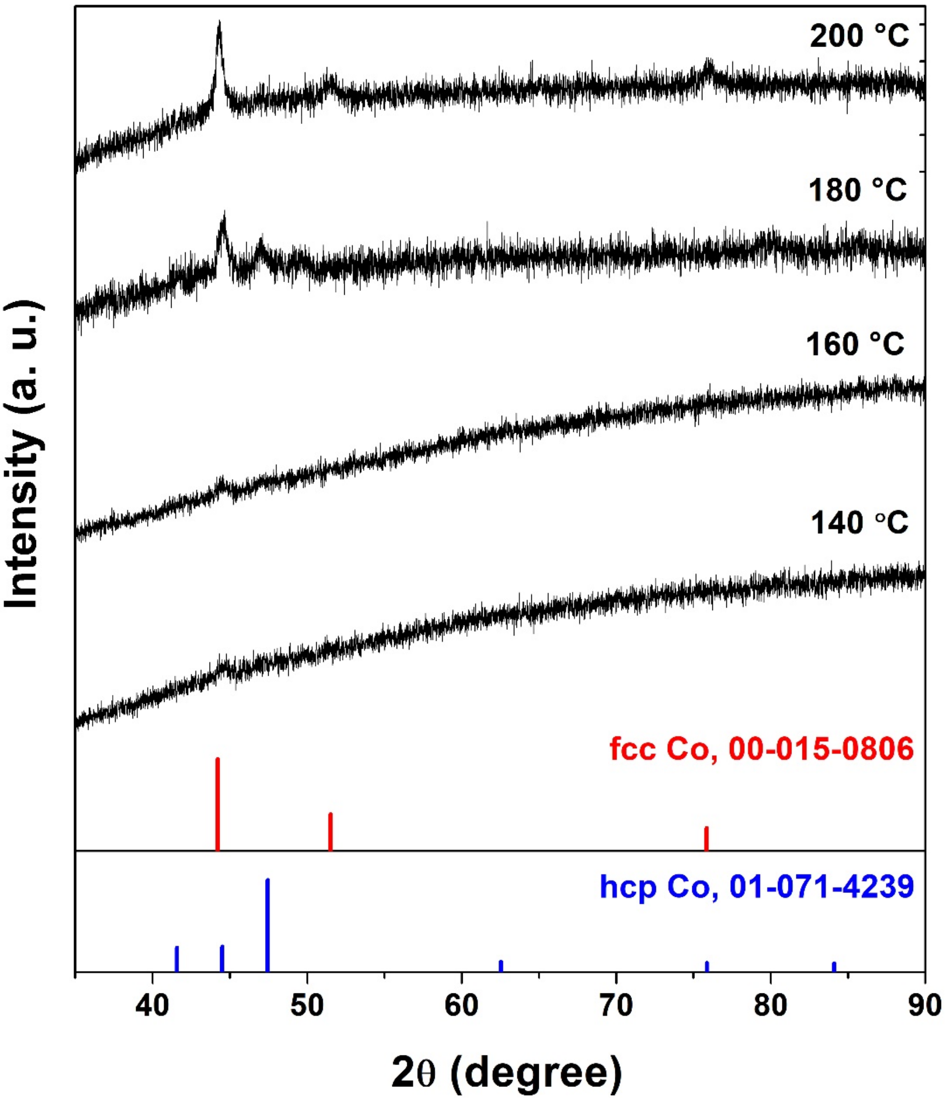

Figure S1. XRD patterns of the temperature-controlled Si-Co-fcc preparation ranging from 140 to 200 °C using 1 mmol $Co(OAc)_2 \cdot 4H_2O$ and 3 mmol phenylsilane under ambient conditions.

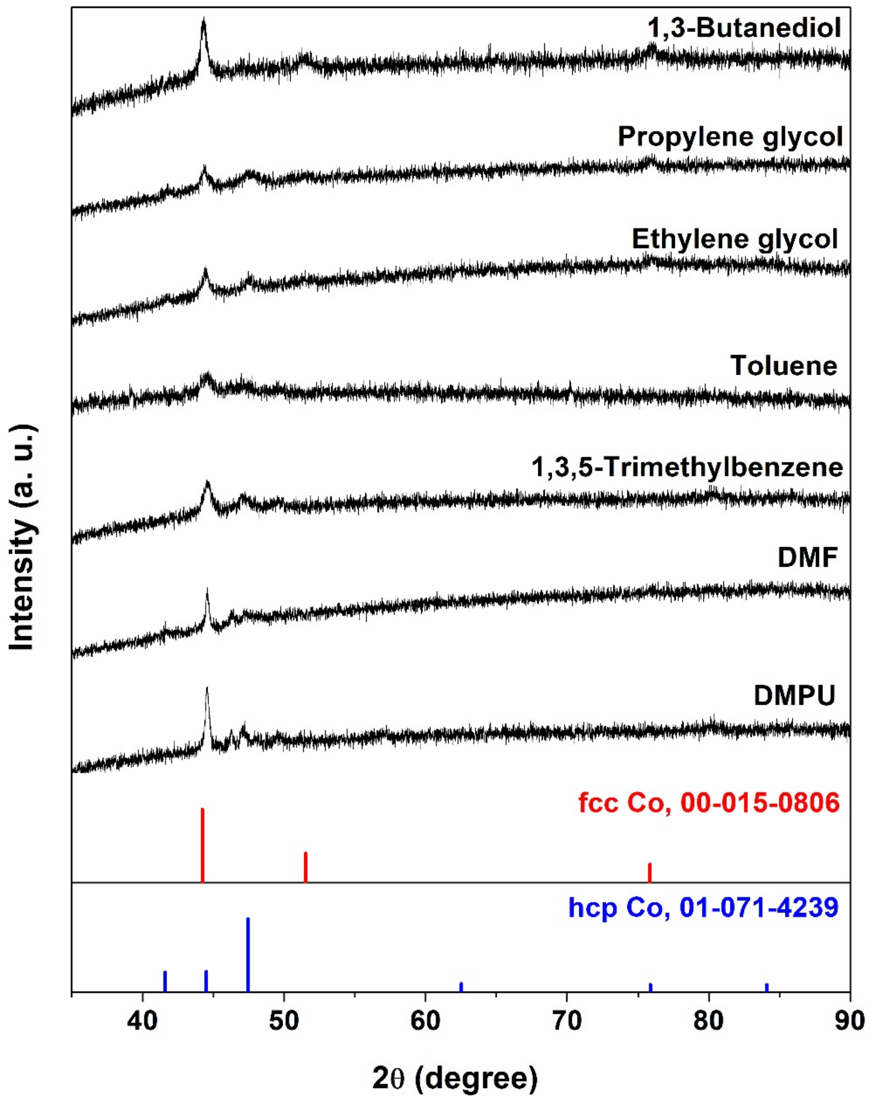

Figure S2. XRD patterns of the Si-Co-fcc preparation controlled by solvent with 1 mmol $Co(OAc)_2{\cdot}4H_2O$ and 3 mmol phenylsilane under 200 °C and Air.

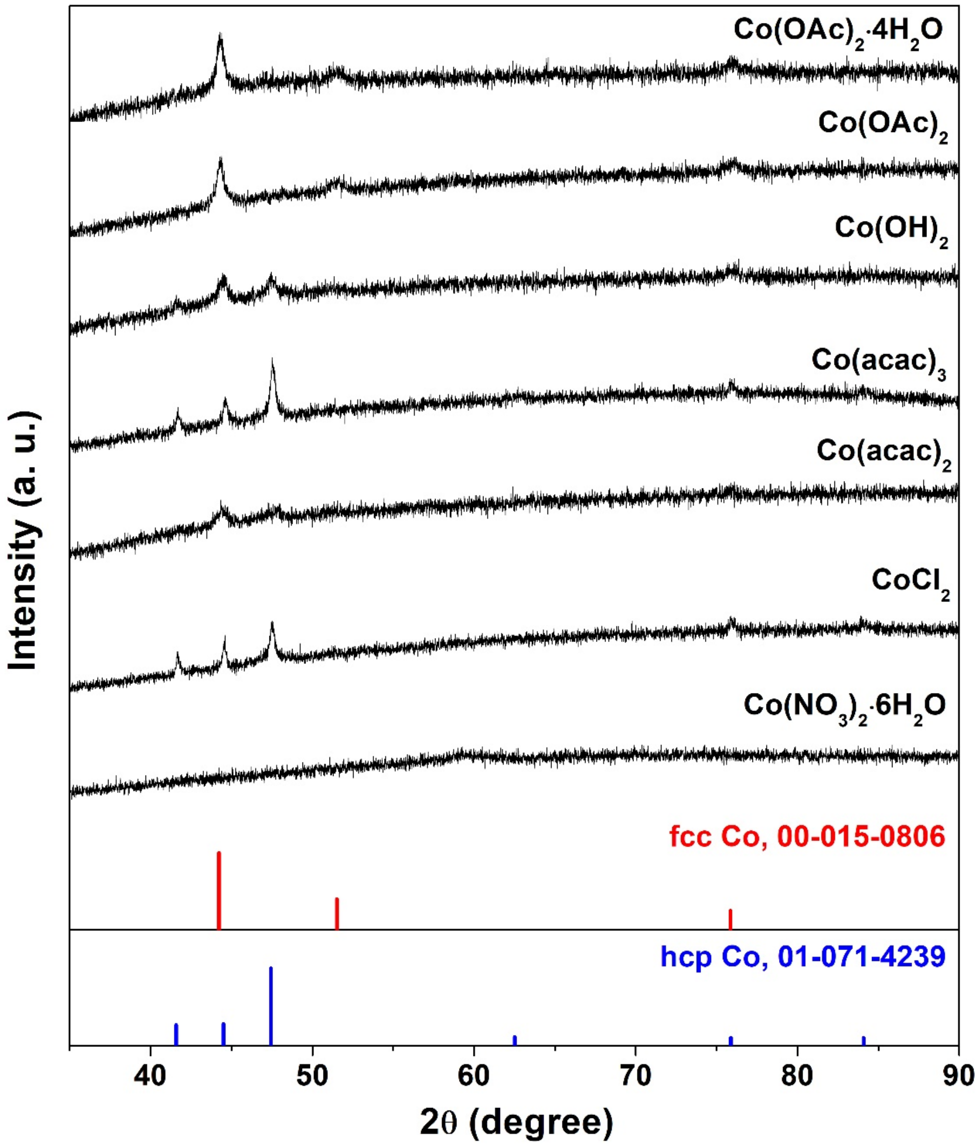

Figure S3. XRD patterns of the Si-Co-fcc preparation controlled by Co source with 3 mmol phenylsilane and 5 mL 1,3-butanediol under 200 °C and Air.

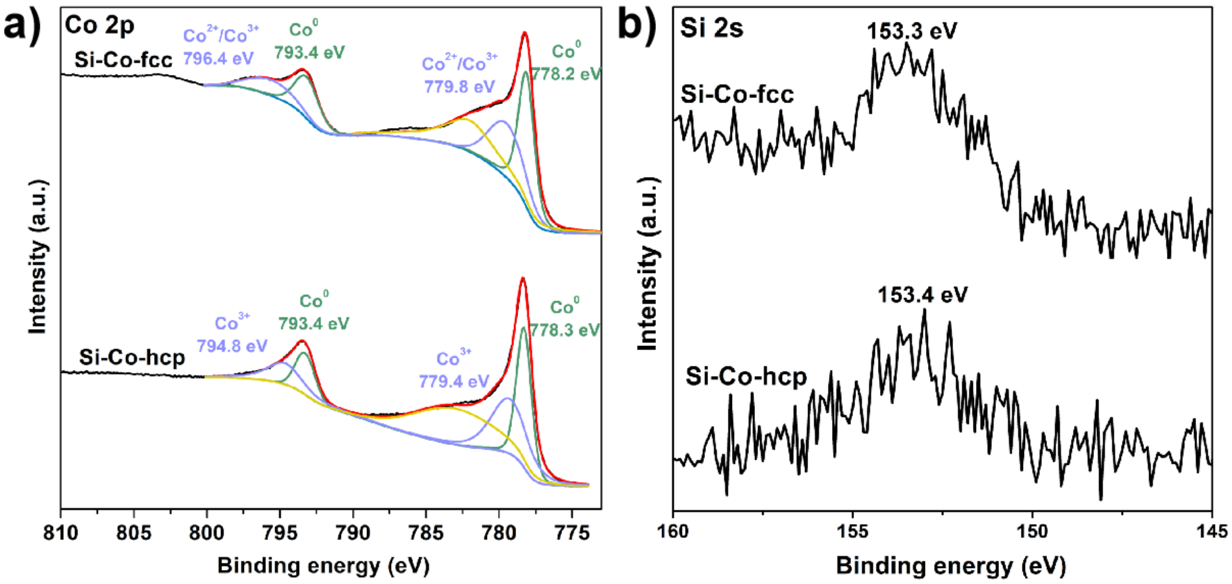

Figure S4. XPS spectra for Si-Co-hcp and Si-Co-fcc without exposure to the atmosphere after H_2 reduction. Reduction conditions: H_2 flow (30 mL/min), 200 °C, 2 h.

The oxidation states of Co were found to be highly similar between Si-Co-hcp and Si-Co-fcc nanoparticles. In the Co 2p region of Si-Co-fcc, the peak at 778.2 eV was ascribed to metallic cobalt, while the peak at approximately 779.8 eV indicated cobalt in the +2/+3 oxidation states.[1,2] Similarly, for Si-Co-hcp, the peak at 778.3 eV was assigned to metallic cobalt, with the peak around 779.4 eV representing cobalt in the +3 oxidation state.[1,3]

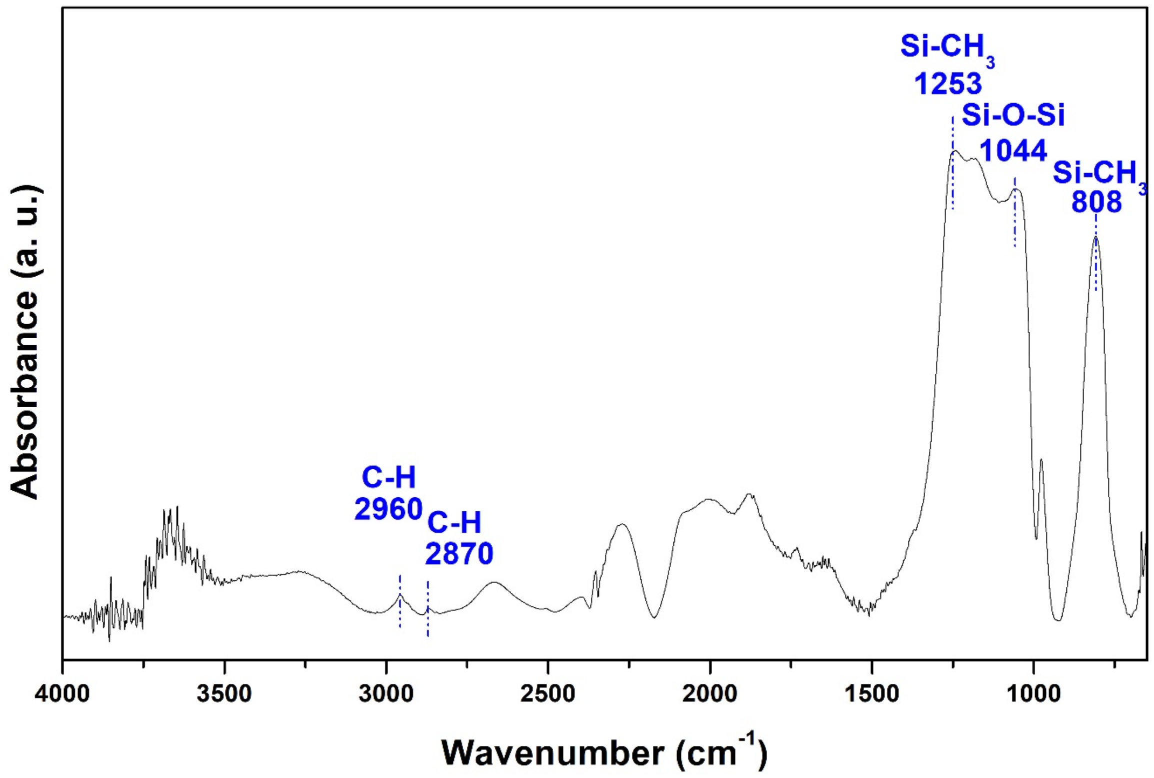

Figure S5. FT-IR of Si-Co-hcp.

The spectrum of the Si-Co-hcp shows peaks at 2960, 2870 cm^{-1} corresponding to vibrations from ν_{as}(C-H) stretching. The peaks at 1253 cm^{-1} corresponding to vibrations from ν_{s}(Si-C) stretching. The peaks at 1044 cm^{-1} correspond to Si-O-Si vibrations. The peaks at 808 cm^{-1} are associated with ν_{as}(Si-C) vibrations.[4,5]

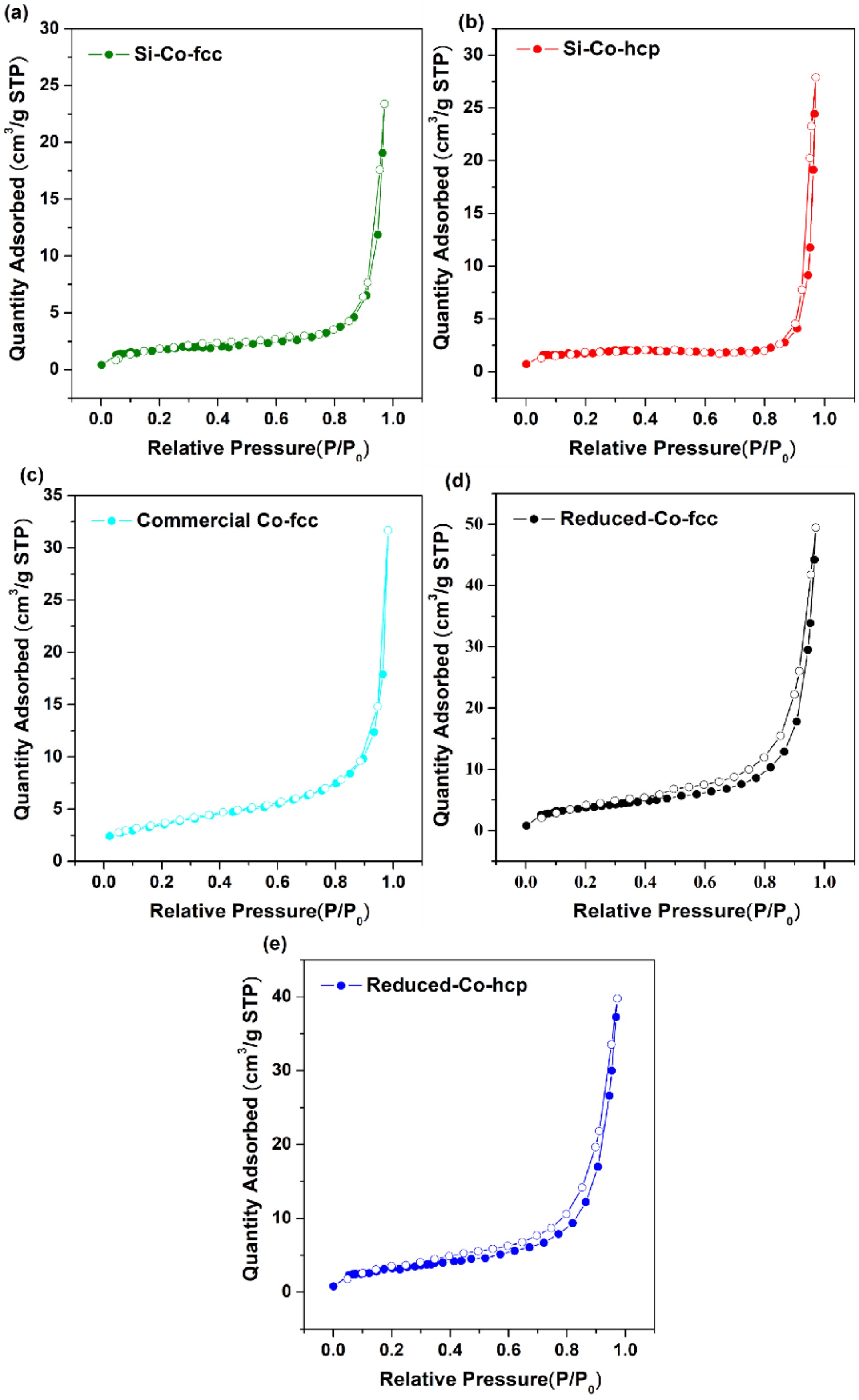

Figure S6. BET surface area analysis results: N_2-adsorption-desorption isotherm plots of (a) Si-Co-fcc, (b) Si-Co-hcp, (c) commercial Co-fcc, (d) reduced-Co-fcc, and (e) reduced-Co-hcp.

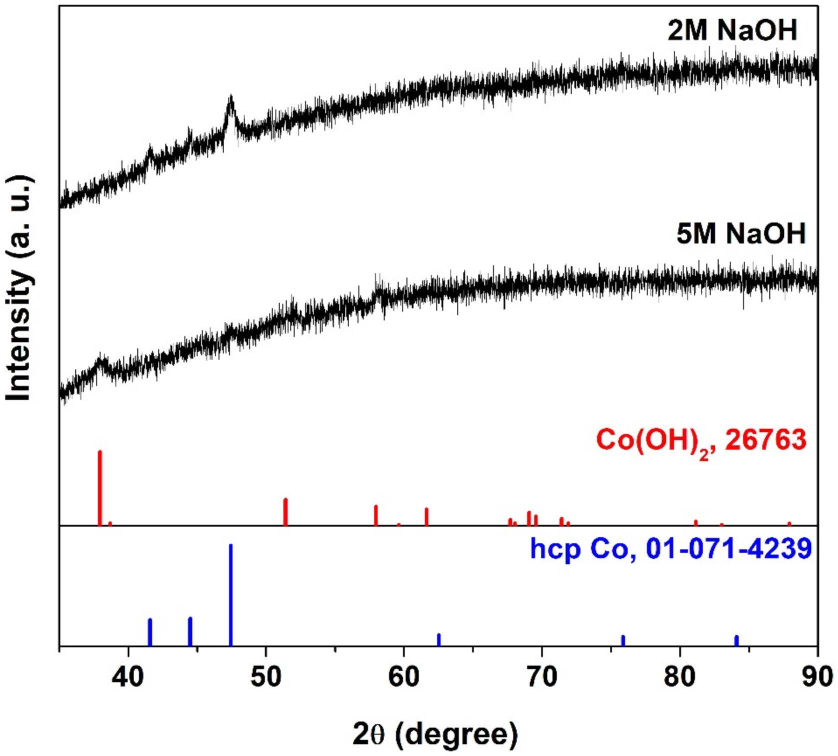

Figure S7. XRD patterns of Si-Co-hcp nanoparticles treated by 5 M and 2 M NaOH in methanol solution after 5 h at room temperature.

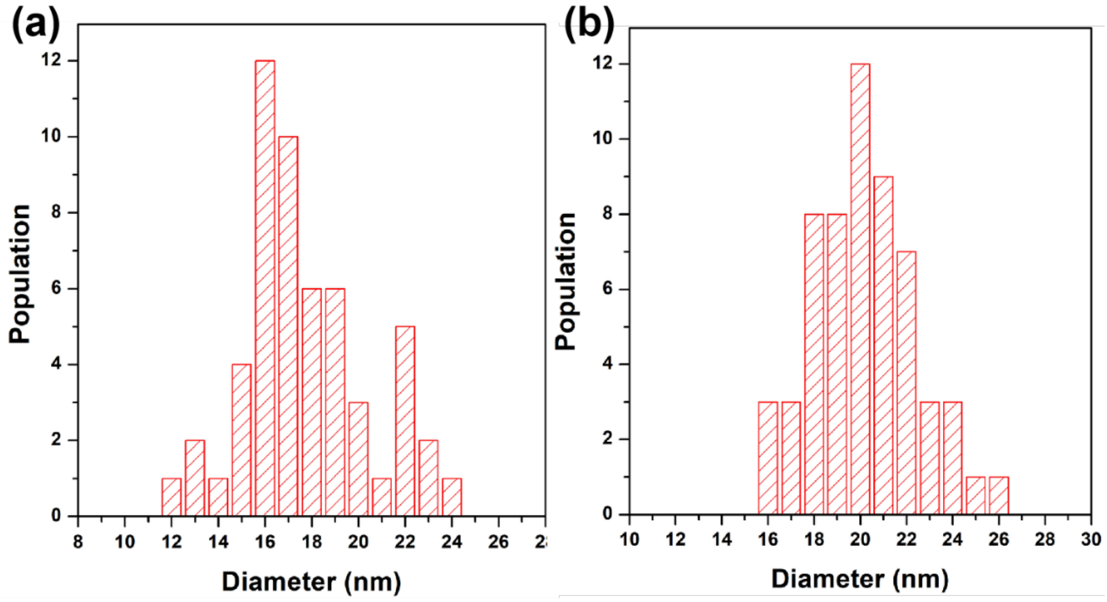

Figure S8. Diameter distribution of Co NPs after NaOH treatment and H_2 reduction: (a) reduced-Co-hcp, (b) reduced-Co-fcc.

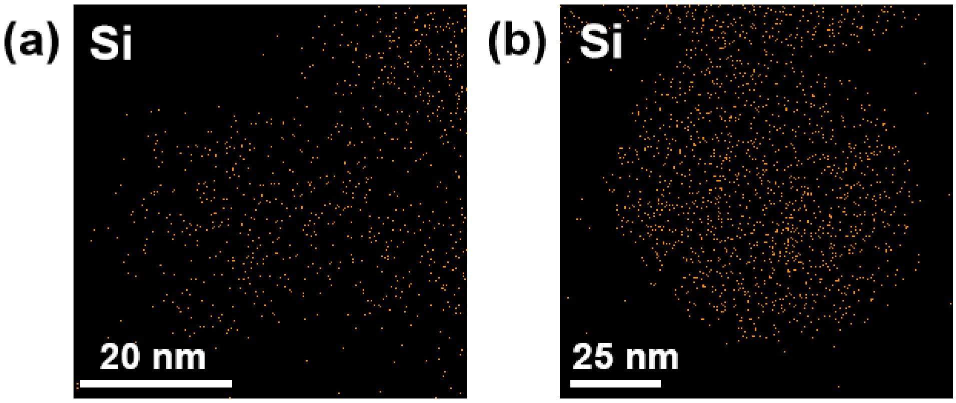

Figure S9. STEM-EDX maps of Co NPs after NaOH treatment and H_2 reduction: (a) reduced-Co-hcp, (b) reduced-Co-fcc.

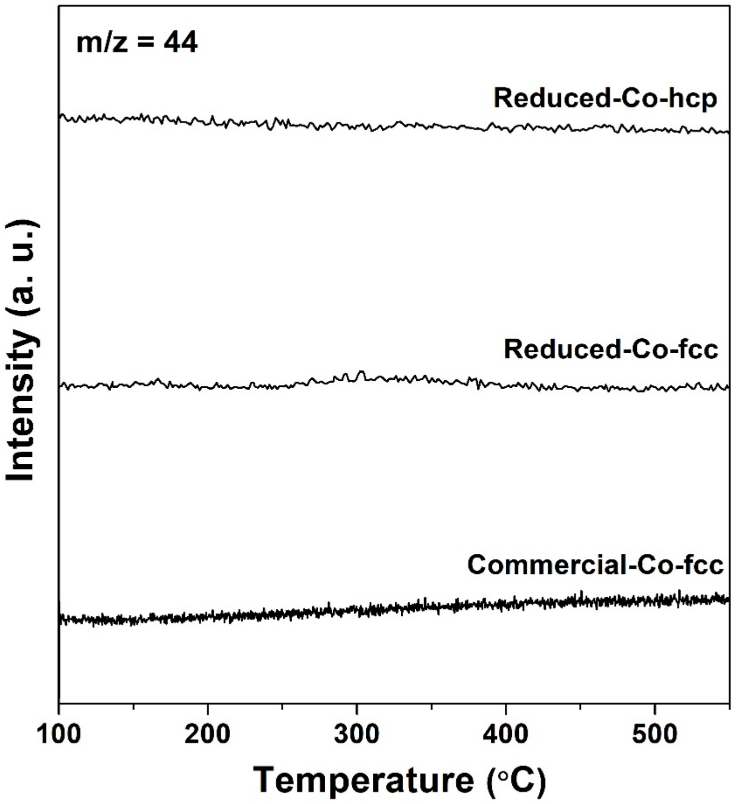

Figure S10. CO_2-TPD for Reduced-Co-hcp, Reduced-Co-fcc and commercial Co-fcc.

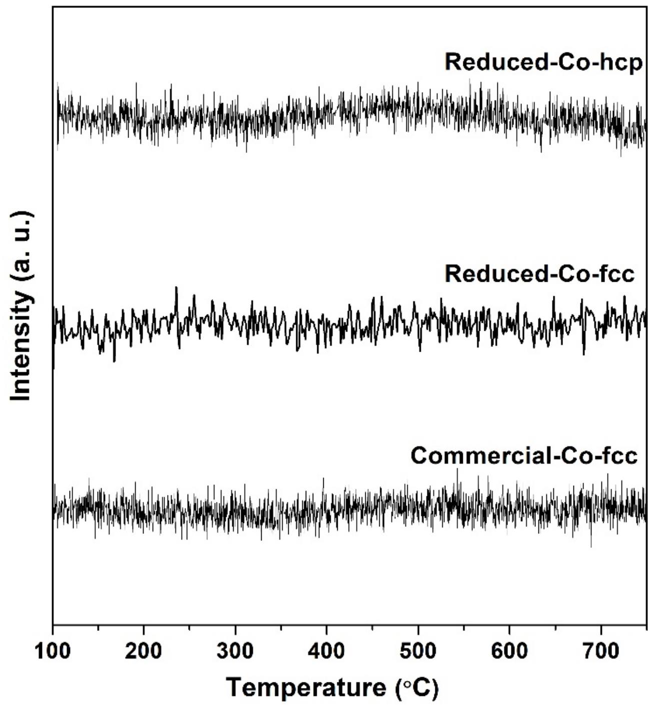

Figure S11. NH_3-TPD for Reduced-Co-hcp, Reduced-Co-fcc and commercial Co-fcc.

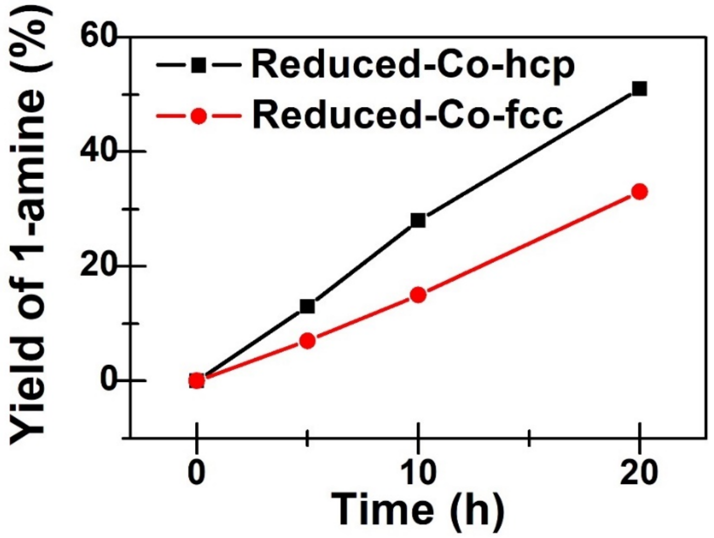

Figure S12. The yield of primary amine in the reductive amination process of benzyldehyde on reduced-Co-hcp or reduced-Co-fcc was investigated under the following conditions: 5 mmol of benzyldehyde, 20 mg of catalyst, 5 mL of toluene, 70 °C, 0.5 MPa of H_2 and 0.4 MPa NH_3.

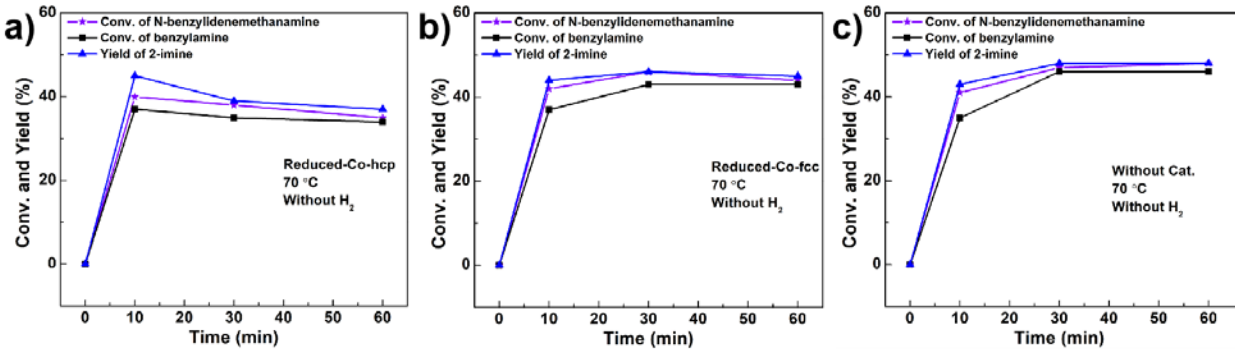

Figure S13. *N*-benzylidenemethanamine hydrogenation in the presence of benzylamine without H_2 on (a) reduced-Co-hcp and (b) reduced-Co-fcc (c) no catalyst was investigated under the following conditions: 5 mmol of *N*-benzylidenemethanamine, 5 mmol of benzylamine, 20 mg of catalyst, 5 mL of toluene, 70 °C.

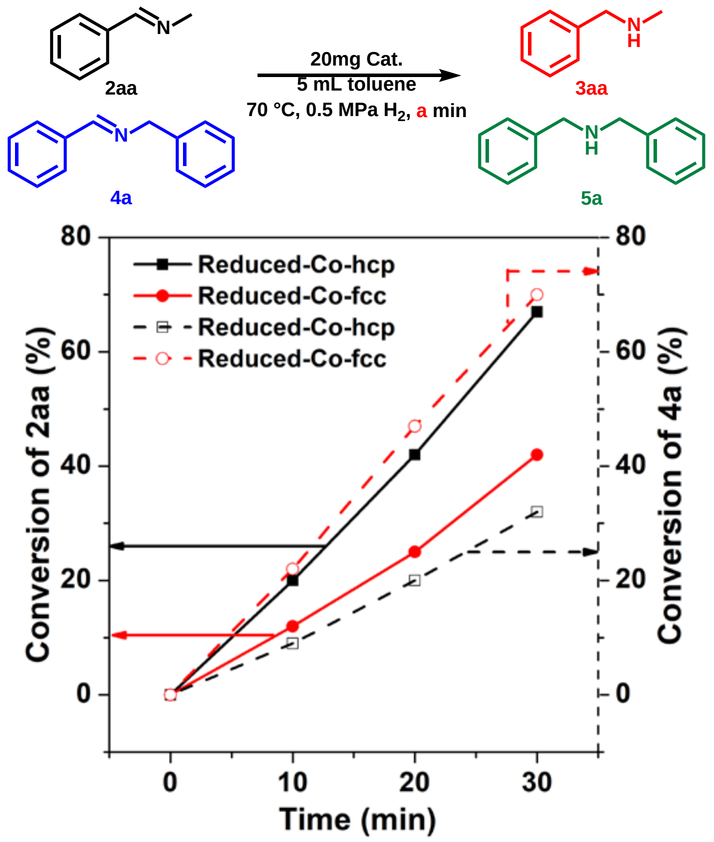

Figure S14. Catalytic performance on equimolar mixture of *N*-benzylidenemethanamine (**2aa**) and *N*-benzylidenebenzylamine (**4a**) as substrates hydrogenation on the reduced-Co-hcp and reduced-Co-fcc. Reaction conditions: **2aa** (0.5 mmol), **4a** (0.5 mmol), catalyst (20 mg), toluene (5 mL), 70 °C, and 0.5 MPa of H_2.

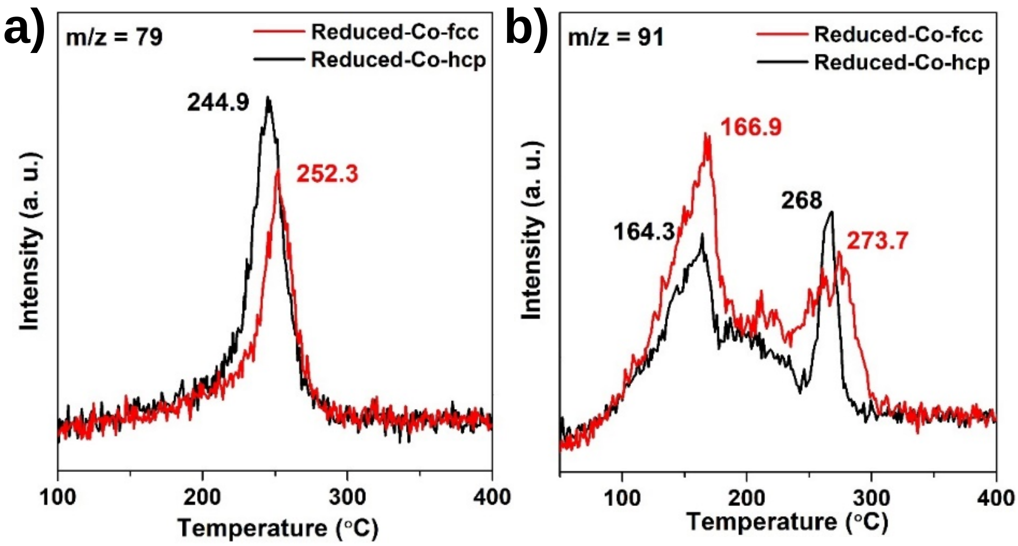

Figure S15. Substrate-TPD on Reduced-Co-hcp and Reduced-Co-fcc. (a) Benzylamine-TPD (**3a**-TPD), (b) *N*-benzylidenebenzylamine-TPD (**4a**-TPD).

The benzylamine (**3a**) exhibits only one desorption peak, whereas the *N*-benzylidenebenzylamine (**4a**) displays two desorption peaks: one at lower temperature, and another at higher temperature.

Desorption energy (E_{de}) is calculated using the Redhead equation[6]:

$$E_{de} = RT_P \left[\ln\left(\frac{\nu_1 T_P}{\beta}\right) - 3.64 \right]$$

Where:

T_p (K) is temperature at the desorption peak maximum.

ν_1 (10^{13} s^{-1}) is the rate constant.

β ($K\cdot s^{-1}$) is the temperature increase rate.

E_{de} ($J\cdot mol^{-1}$) is desorption energy.

Since adsorption and desorption are inverse processes, the adsorption energy E_{ad} is calculated as:

$$E_{ad} = -E_{de}$$

To express the adsorption energy of benzylamine (**3a**) over Reduced-Co-hcp, we use $E_{ad\_3a\_hcp}$. Similarly, $E_{ad\_3a\_fcc}$, $E_{ad\_4a\_hcp\_1}$, $E_{ad\_\_4a\_fcc\_1}$, $E_{ad\_4a\_hcp\_2}$, $E_{ad\_4a\_fcc\_2}$ represent the adsorption energies of **3a** on Reduced-Co-fcc and **4a** on Reduced-Co-hcp and Reduced-Co-fcc for the left peaks (1) and right peaks (2) in Figure SE.

Calculated adsorption energies: $E_{ad\_3a\_hcp} = -151.5$ kJ·mol^{-1}, $E_{ad\_3a\_fcc} = -153.8$ kJ·mol^{-1}, $E_{ad\_4a\_hcp\_1} = -127.5$ kJ·mol^{-1}, $E_{ad\_4a\_fcc\_1} = -128.1$ kJ·mol^{-1}, $E_{ad\_4a\_hcp\_2} = -158.5$ kJ·mol^{-1}, $E_{ad\_4a\_fcc\_2} = -160.2$ kJ·mol^{-1}.

In conclusion, there is no significant difference in adsorption energy of 3a and 4a between Reduced-Co-hcp and fcc.

Table S1. Reaction conditions for synthesis of fcc and hcp NPs.[a]

Entry	Co source	Amount of phenylsilane (mmol)	Solvent (5mL)	T. (°C)	Crystal Phase
1	$Co(OAc)_2{\cdot}4H_2O$	3	Propylene glycol	200	fcc + hcp
2	$Co(OAc)_2{\cdot}4H_2O$	3	Ethylene glycol	200	fcc + hcp
3	$Co(OAc)_2{\cdot}4H_2O$	3	Toluene	200	fcc + hcp
4	$Co(OAc)_2{\cdot}4H_2O$	3	1,3,5-Trimethylbenzene	200	fcc + hcp
5	$Co(OAc)_2{\cdot}4H_2O$	3	DMF	200	fcc + hcp
6	$Co(OAc)_2{\cdot}4H_2O$	3	DMPU	200	fcc + hcp

[a] All synthesis were run for 1 hour.

Table S2. Atomic concentration of Co 2p to Si 2s in Si-Co-fcc and Si-Co-hcp.

Catalysts	Peak	Atomic Conc.%
Si-Co-fcc	Si 2s	44.7
	Co 2p	55.3
Si-Co-hcp	Si 2s	14.9
	Co 2p	85.1

Table S3. The surface and structure parameters of hydrosliane-assisted method synthesized Si-Co-hcp and Si-Co-fcc nanoparticles, the base treated Co nanoparticles and the commercial Co-fcc nanoparticles, and the cobalt nanoparticles in the reported literatures.

Sample	Crystal phase	Average size observed (nm)	Crystallite diameter [a] (nm)	Calculated surface area [b] (m^2 g^{-1})	BET surface area (m^2 g^{-1})	Ref.
Si-Co-hcp	hcp	20	15.9	42.3	6	This work
Si-Co-fcc	fcc	23	20.6	32.8	6	This work
Reduced-Co-hcp	hcp	16	18.2	37.0	11	This work
Reduced-Co-fcc	fcc	20	22.5	29.9	13	This work
Reduced-commercial Co-fcc	fcc	-	27.4	24.6	12	This work
HCP-Co	hcp	-	28.2	23.9	10	[7]
Co nanoparticles prepared by one-pot synthesis using oleic acid	hcp	9.2	-	-	-	[8]
Co nanoparticles prepared by reduction with hydrazine in ethanol/alkaline medium	Mix phase (hcp and fcc)	200-230	-	-	-	[9]
Co nanoparticles prepared by plasma discharge and ultrasonic treatment	Mix phase (hcp and fcc)	8.3	-	-	-	[10]

[a] derived by the Scherrer equation from XRD measurements.

[b] assuming Co nanoparticles are spherical particles.

The calculation formula of the specific surface area:

$$S_A = \frac{S_1}{m_1} = \frac{S_1}{\rho V_1} = \frac{\pi \times D \times D}{\rho \times \frac{1}{6} \times \pi \times D \times D \times D} = \frac{6}{\rho D} = \frac{6}{8.9 g/cm^3 \times D}$$

S_A means the specific surface area of one cobalt atom.

S_1 means the surface area of one cobalt atom.

m_1 means the weight of one cobalt atom.

V_1 means the volume of one cobalt atom.

D means the diameter of one cobalt atom. Diameter is derived by the Scherrer equation from XRD measurements.

ρ means the density of cobalt, $8.9 g/cm^3$.

Table S4. The atomic concentration of Si and Co element in Si-Co-hcp, Si-Co-fcc, reduced-Co-hcp and reduced-Co-fcc through STEM-EDS analysis.

**Cat.**	**Atomic Concentration (%)**	
	Si	Co
Si-Co-hcp	0.30	99.7
Si-Co-fcc	0.88	99.1
Reduced-Co-hcp	0.19	99.8
Reduced-Co-fcc	0.36	99.6

Table S5. The hydrogenation of 1a over Co-fcc catalysts. [a]

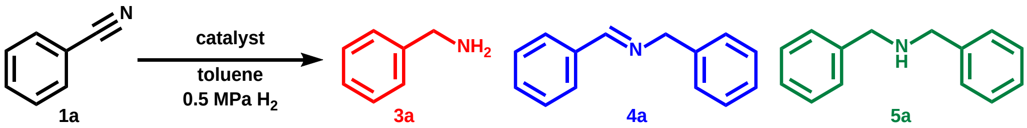

Entry	Cat.	Temp. (°C)	Time (h)	Conv.[b] (%)	Yield[b] (%)		
					**3a**	**4a**	**5a**
1	Reduced-Co-fcc	70	2.5	42	21	23	0
2	Reduced-commercial Co-fcc	70	2.5	46	23	24	0

[a] Reaction conditions: catalyst (20 mg), **1a** (0.5 mmol), toluene (5 mL), pH_2 (0.5 MPa), 2.5 h.

[b] Determined by GC.

Table S6. Comparison of primary amine selectivity with literature reports in nitrile hydrogenation over Co catalyst.

Catalyst	Substrate	Ammonia	Solvent	pH_2 (MPa)	Temp. (°C)	Time (h)	Yield (%)	Ref.
Reduced-Co-hcp	benzonitrile	none	toluene	0.5	70	20	97	This work
Co/SiO_2	benzonitrile	none	cyclohexane	0.5	50	20	78	[11]
Co/SiO_2	benzonitrile	none	cyclohexane/DMF	1	100	6	89	[11]
Co_2P NR/HT	valeronitrile	none	2-propanol	4	150	2	90	[12]
$CoBr_2$ + $NaBHEt_3$	benzonitrile	none	THF	2	110	4	83	[13]
Zr_{12}-TPDC-Co	benzonitrile	none	toluene	4	110	42	100	[14]
Co_2P/HT	valeronitrile	NH_3 aq. (32 equiv.)	2-propanol	4	150	1	99	[15]
$Co(OAc)_2$ + Zn	benzonitrile	NH_3 aq. (1 equiv.)	methanol	4	120	15	94	[16]
Co-B_{12}@CeO_2	benzonitrile	NH_3 aq. (10 equiv.)	2-propanol	3	120	15	96	[17]
Co-MOF@C-800	benzonitrile	pNH_3 (0.5 MPa)	toluene	2.5	120	16	97	[18]
$Co(OAc)_2$/Phen @Al_2O_3	heptanenitrile	NH_3 aq. (8 equiv.)	2-propanol	4	85	24	98	[19]
Co-N-C@MgO-700	benzonitrile	NH_3 aq. (5.3 equiv.)	2-propanol	2	80	24	98	[20]

Table S7. Hydrogenation of 2-methoxylbenzonitrile over reduced-Co-hcp.

Entry	T (˚C)	Time (h)	Conv. (%)	Yields (%)		
				3c	3d	3e
1	50	0.5	33	33	0	0
2	60	4.0	>99	44	0	56
3	70	1.3	>99	59	0	41
4	90	1.3	>99	53	0	47

Table S8. Adsorption over reduced-Co-hcp and reduced-Co-fcc.

**Cat.**	**Benzylamine reduced ratio (%)** [a]	**N-benzylidenebenzylamine reduced ratio (%)** [a]
Reduced-Co-hcp	1	0
Reduced-Co-fcc	12	26

[a] The reduced ratio is determined by subtracting the initially added amount of the organic compound from the quantity adsorbed, as measured by GC, after the completion of a 1-hour period.

The chlorobenzene adsorption was carried out using the same process, excluding the addition of N-containing organic compounds, for a duration of 1 hour, with toluene serving as the internal standard. Consequently, the adsorption quantities of chlorobenzene over reduced-Co-hcp and reduced-Co-fcc nanoparticles were comparable, and the reduced ratios of chlorobenzene were both 1%.

3. NMR results

2-methylbenzylamine (**3b**)[21]

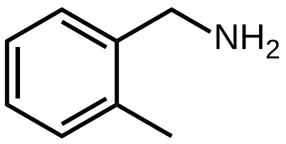

3b

Light yellow solid. Yield: 97%. ^{1}H NMR (400 MHz, $CDCl_3$) δ 7.30 (d, *J* = 7.0 Hz, 1H), 7.22-7.17 (m, 1H), 7.16 (d, *J* = 3.9 Hz, 2H), 3.86 (s, 2H), 2.34 (s, 3H), 1.49 (s, 2H); ^{13}C NMR (101 MHz, $CDCl_3$) δ 141.1, 135.5, 130.3, 126.9, 126.2, 44.1, 18.8 ppm.

4-methylbenzylamine (**3c**)[21]

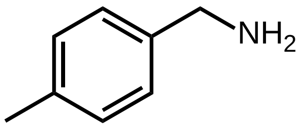

3c

Light yellow solid. Yield: 85%. ^{1}H NMR (400 MHz, $CDCl_3$) δ 7.20 (d, *J* = 7.8 Hz, 1H), 7.14 (d, *J* = 7.8 Hz, 1H), 3.82 (s, 1H), 2.34 (s, 2H), 1.54 (s, 1H); ^{13}C NMR (101 MHz, $CDCl_3$) δ 140.4, 136.4, 129.2, 127.0, 46.2, 21.0 ppm.

2-methoxybenzylamine (**3d**)[22]

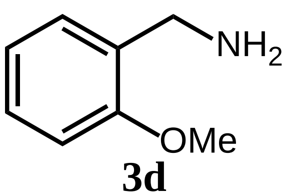

3d

Colorless oil. Yield: 82 %. ^{1}H NMR (400 MHz, $CDCl_3$) δ 7.23 (m, 2H), 6.90 (m, 2H), 3.85 (s, 3H), 3.82 (s, 2H), 1.65 (s, 4H); ^{13}C NMR (101 MHz, $CDCl_3$) δ 157.5, 131.1, 128.5, 128.1, 120.6, 110.3, 55.2, 42.6 ppm.

4-methoxybenzylamine (**3e**)[23]

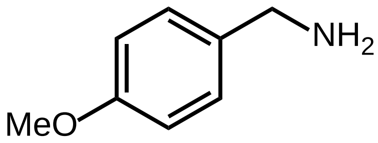

3e

White solid. Yield: 96 %. ^{1}H NMR (400 MHz, $CDCl_3$) 7.23 (d, *J* = 8.5 Hz, 2H), 6.87 (d, *J* = 11.5 Hz, 2H), 3.80 (s, 5H), 1.49 (s, 2H); ^{13}C NMR (101 MHz, $CDCl_3$) δ 158.5, 135.6, 128.3, 113.9, 55.3, 45.9 ppm.

ethyl 4-(aminomethyl)benzoate (**3f**)[24]

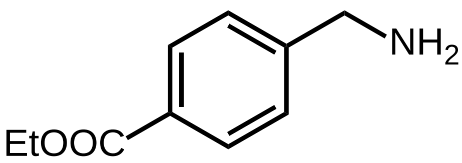

3f

White solid. Yield: 97%. ^{1}H NMR (400 MHz, $CDCl_3$) δ 8.01 (d, *J* = 8.0 Hz, 2H), 7.38 (d, *J* = 7.8 Hz, 2H), 4.37 (m, 2H), 3.93 (s, 2H), 1.53 (s, 2H), 1.37 (s, 3H); ^{13}C NMR (101 MHz, $CDCl_3$) δ 166.5, 148.3, 129.8, 129.1, 128.1, 127.8, 126.9, 60.8, 46.2, 14.3 ppm.

4-iodobenzyl amine (**3g**)[25]

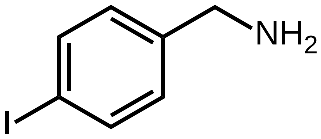

3g

White solid. Yield: 96 %. ^{1}H NMR (400 MHz, $CDCl_3$) δ 7.73 (m, 2H), 7.20 (t, *J* = 84.3 Hz, 2H), 3.81 (s, 2H), 1.57 (s, 2H); ^{13}C NMR (101 MHz, $CDCl_3$) δ 142.9, 137.6, 129.1, 91.8, 45.9 ppm.

4-(aminomethyl)pyridine (**3h**)[26]

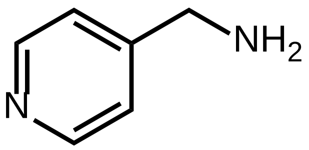

3h

Yellow liquid. Yield: 71%. ^{1}H NMR (400 MHz, $CDCl_3$) δ 8.54 (m, 2H), 7.27 (dd, J = 13.6, 5.7 Hz, 2H), 3.87 (d, J = 29.8 Hz, 2H), 1.90 (s, 2H). ^{13}C NMR (101 MHz, $CDCl_3$) δ 151.8, 149.8, 148.9, 122.9, 122.0, 51.8, 45.2 ppm.

Di(4-pyridylmethyl)amine (**4h**)[27]

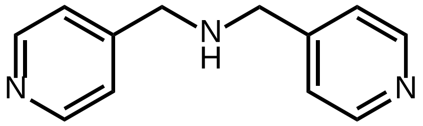

4h

Yellow oil. Yield: 28 %. ^{1}H NMR (400 MHz, $CDCl_3$) δ 8.57 (dd, J = 4.6, 1.4 Hz, 4H), 7.29 (m, 4H), 3.84 (s, 4H). ^{13}C NMR (101 MHz, $CDCl_3$) δ 149.93, 148.91, 122.93, 51.89 ppm.

Phenethylamine (**3i**)[28]

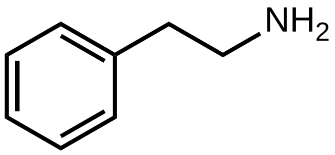

3i

Yellow solid. Yield: 96 %. ^{1}H NMR (400 MHz, $CDCl_3$) δ 7.30 (t, *J* = 7.4 Hz, 2H), 7.20 (t, *J* = 7.2 Hz, 3H), 2.97 (t, *J* = 6.9 Hz, 2H), 2.75 (t, *J* = 6.9 Hz, 2H), 1.39 (s, 2H). ^{13}C NMR (101 MHz, $CDCl_3$) δ 139.7, 128.8, 128.5, 126.2, 43.5, 40.1 ppm.

n-Octylamine (**3j**)[22]

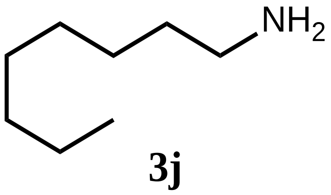

3j

Colorless oil. Yield: 96 %.^{1}H NMR (400 MHz, $CDCl_3$) δ 2.68 (t, *J* = 7.0 Hz, 2H), 1.75 (s, 2H), 1.44 (m, 2H), 1.29 (s, 10H), 0.88 (t, *J* = 6.8 Hz, 3H). ^{13}C NMR (101 MHz, $CDCl_3$) δ 41.1, 31.8, 31.6, 29.3, 26.9, 22.6, 14.0 ppm.

^{1}H NMR of 2-methylbenzylamine (3b)

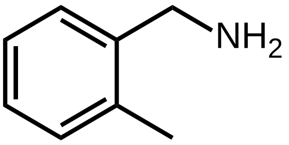

3b

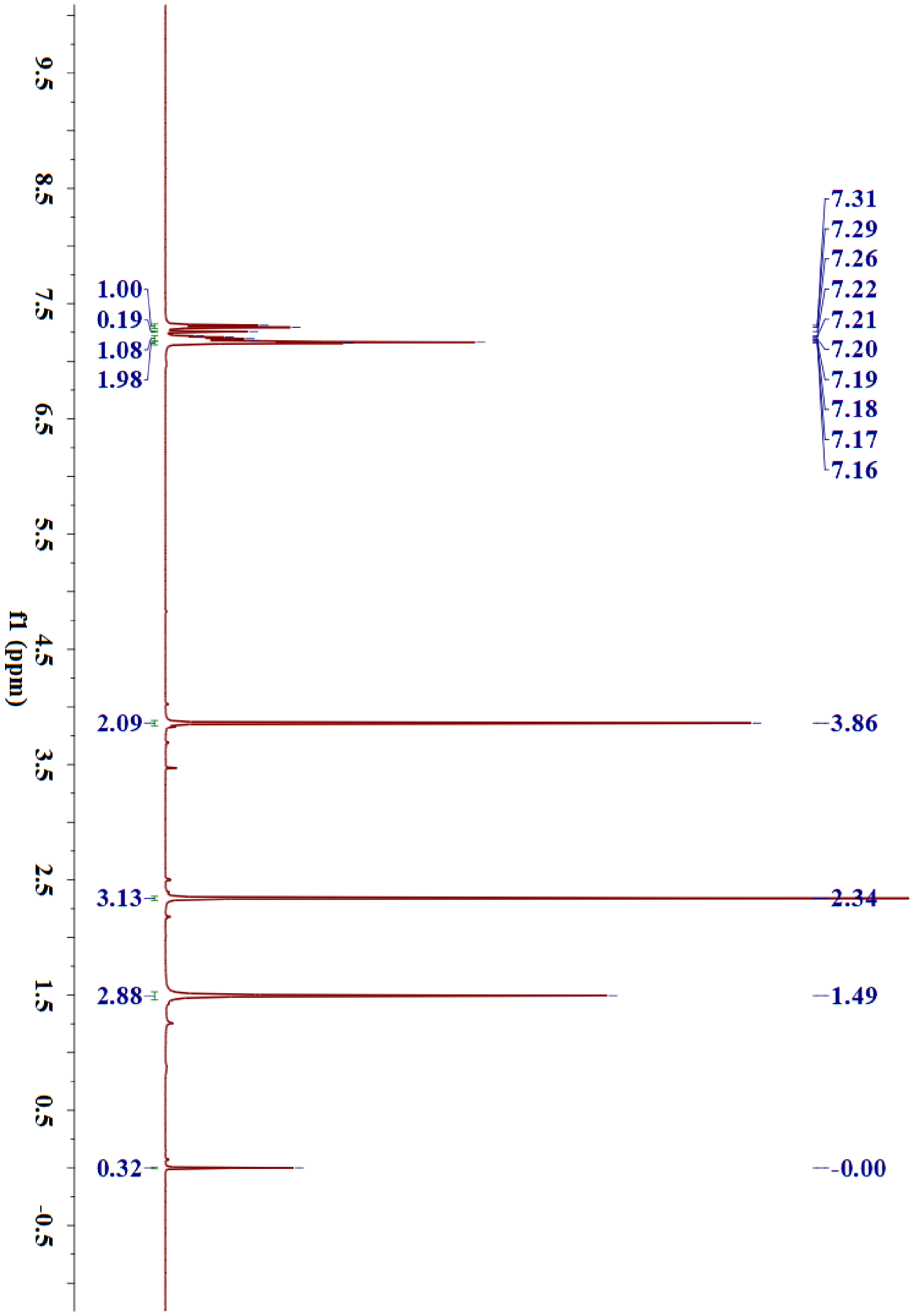

^{13}C NMR of 2-methylbenzylamine (3b)

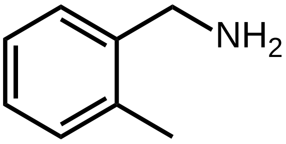

3b

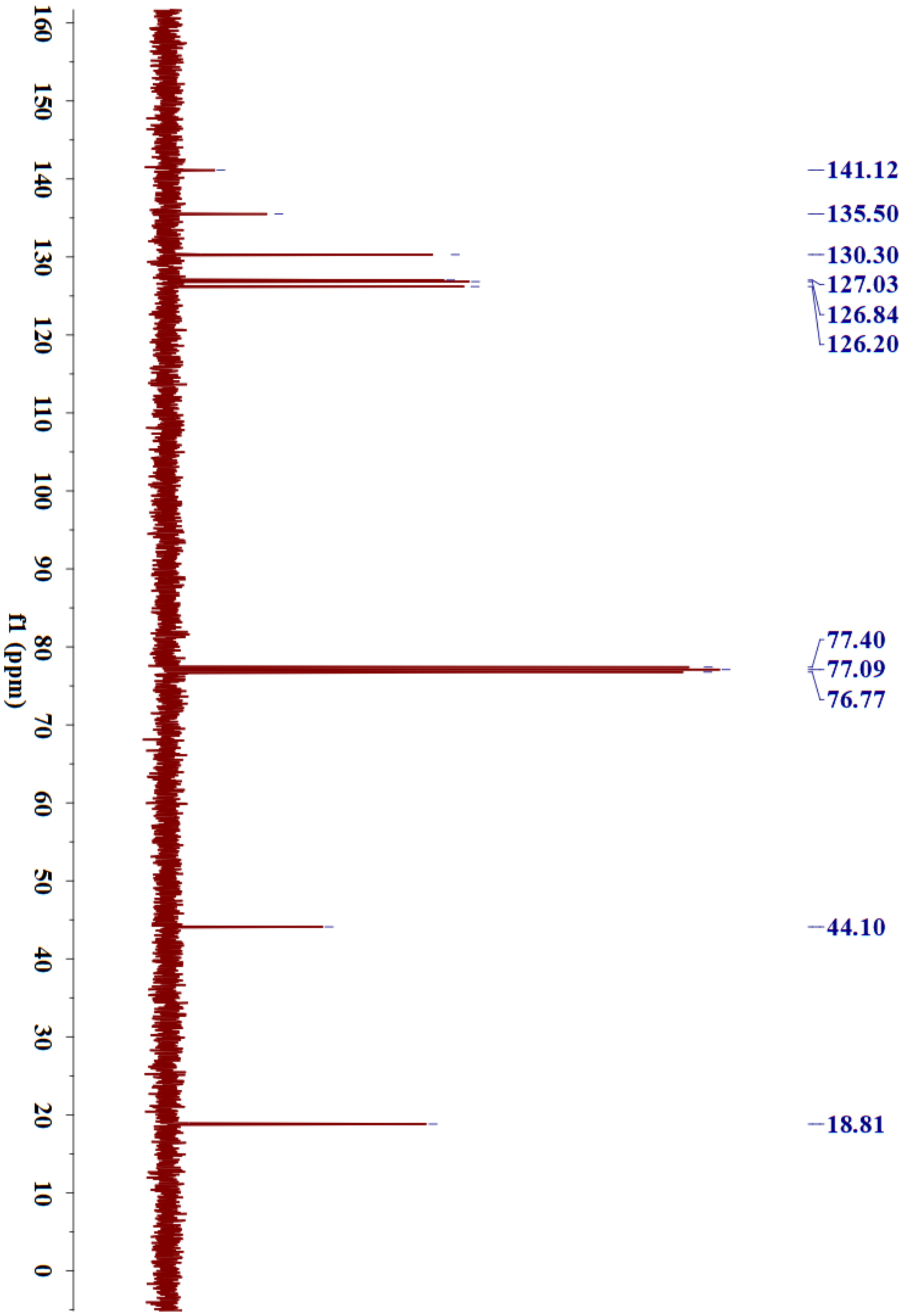

^{1}H NMR of 4-methylbenzylamine (3c)

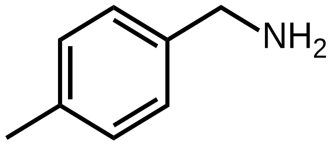

3c

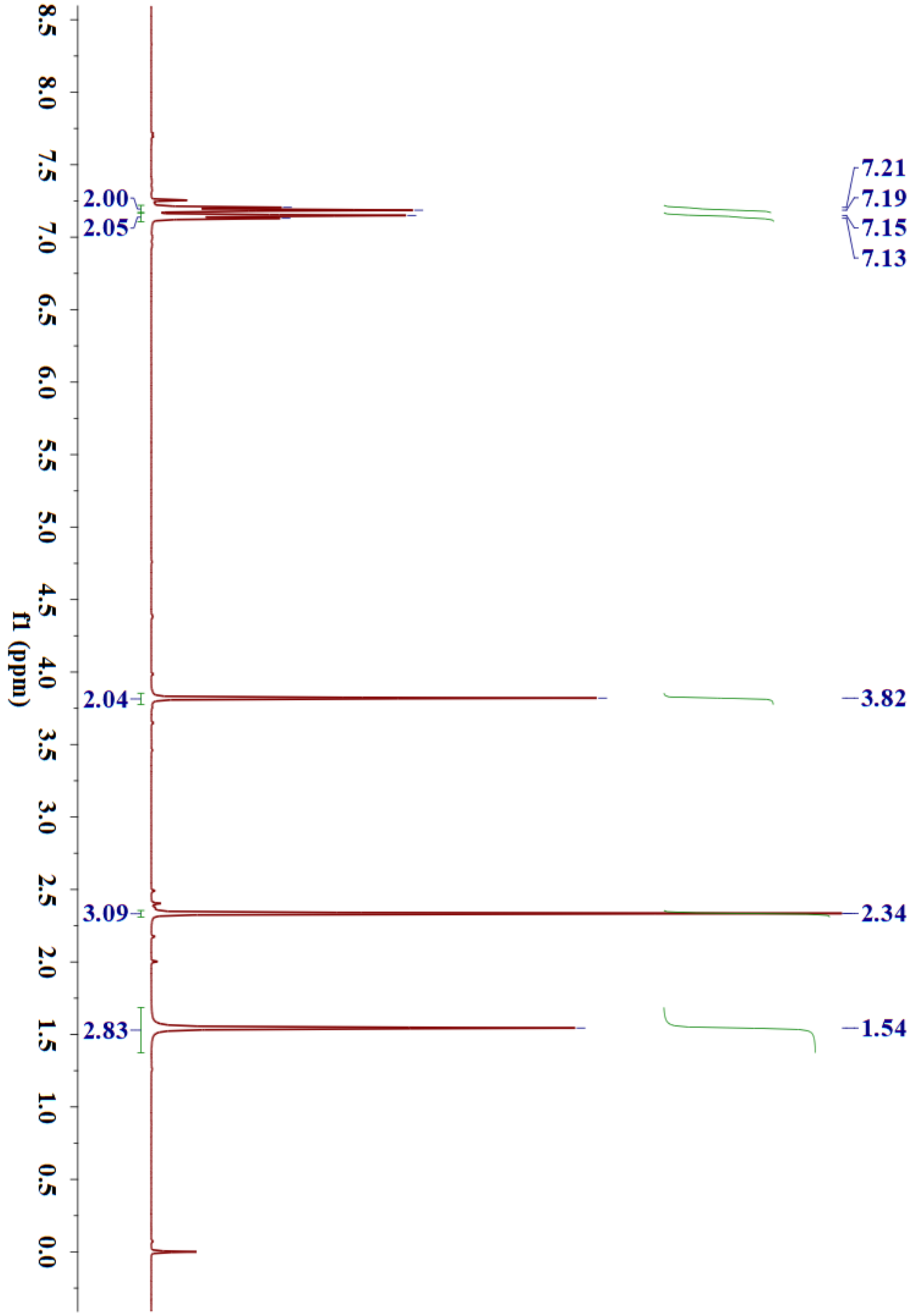

^{13}C NMR of 4-methylbenzylamine (3c)

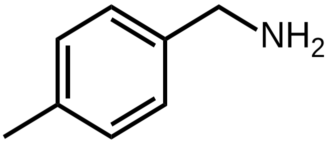

3c

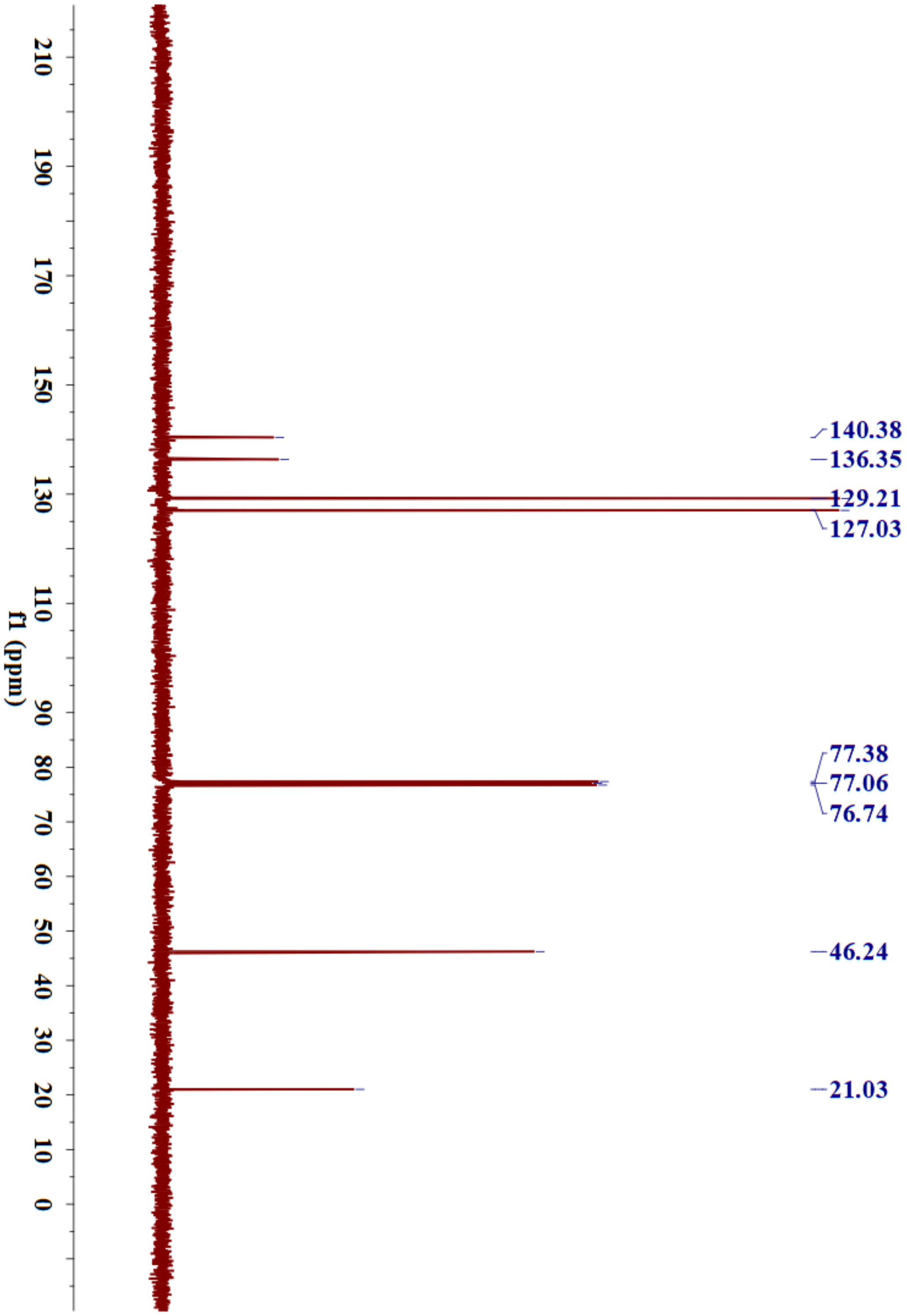

^{1}H NMR of 2-methoxybenzylamine (3d)

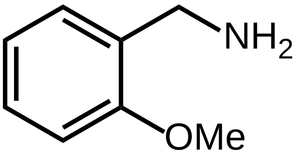

3d

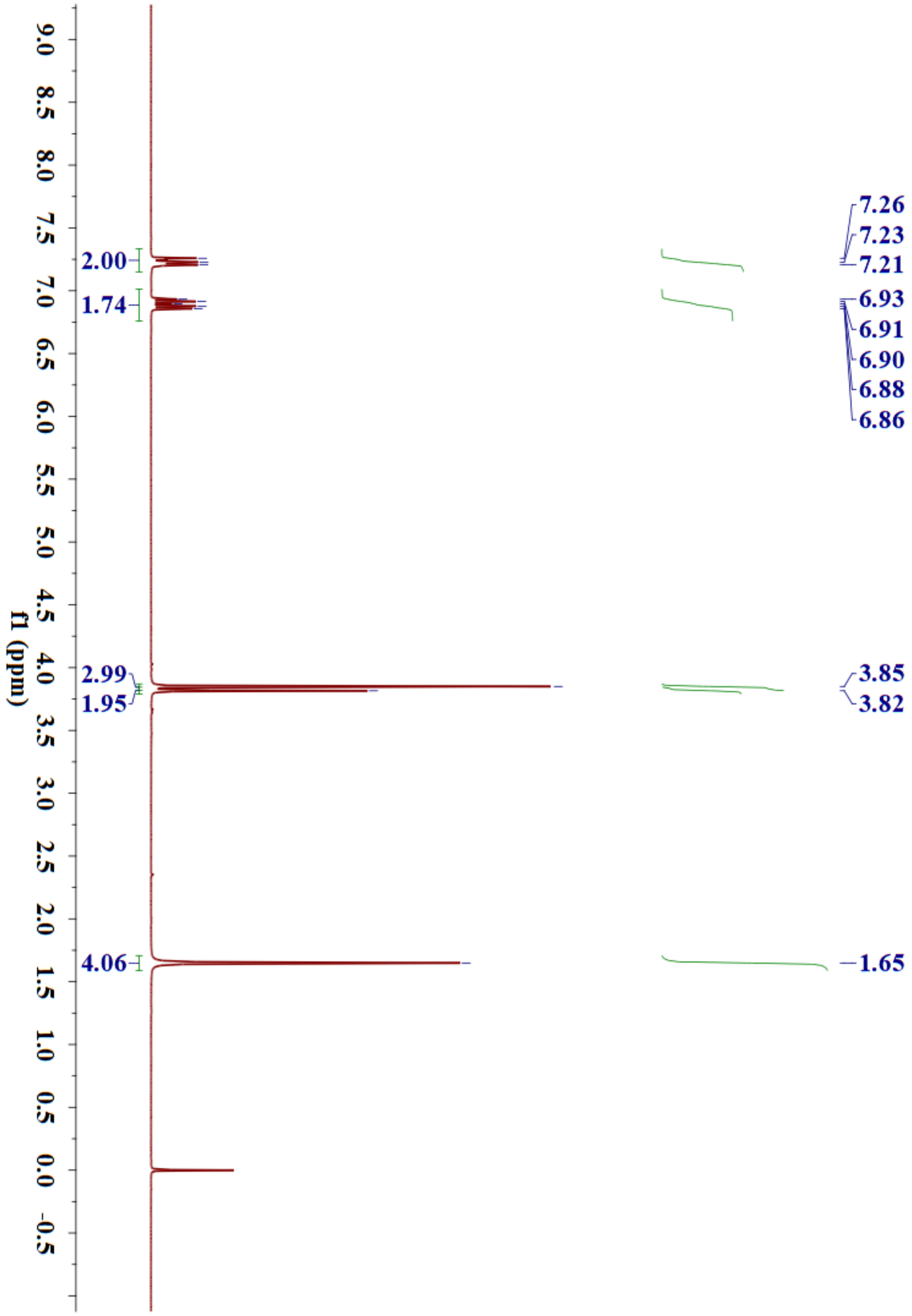

^{13}C NMR of 2-methoxybenzylamine (3d)

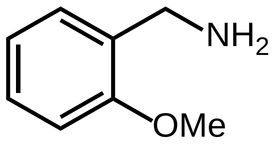

3d

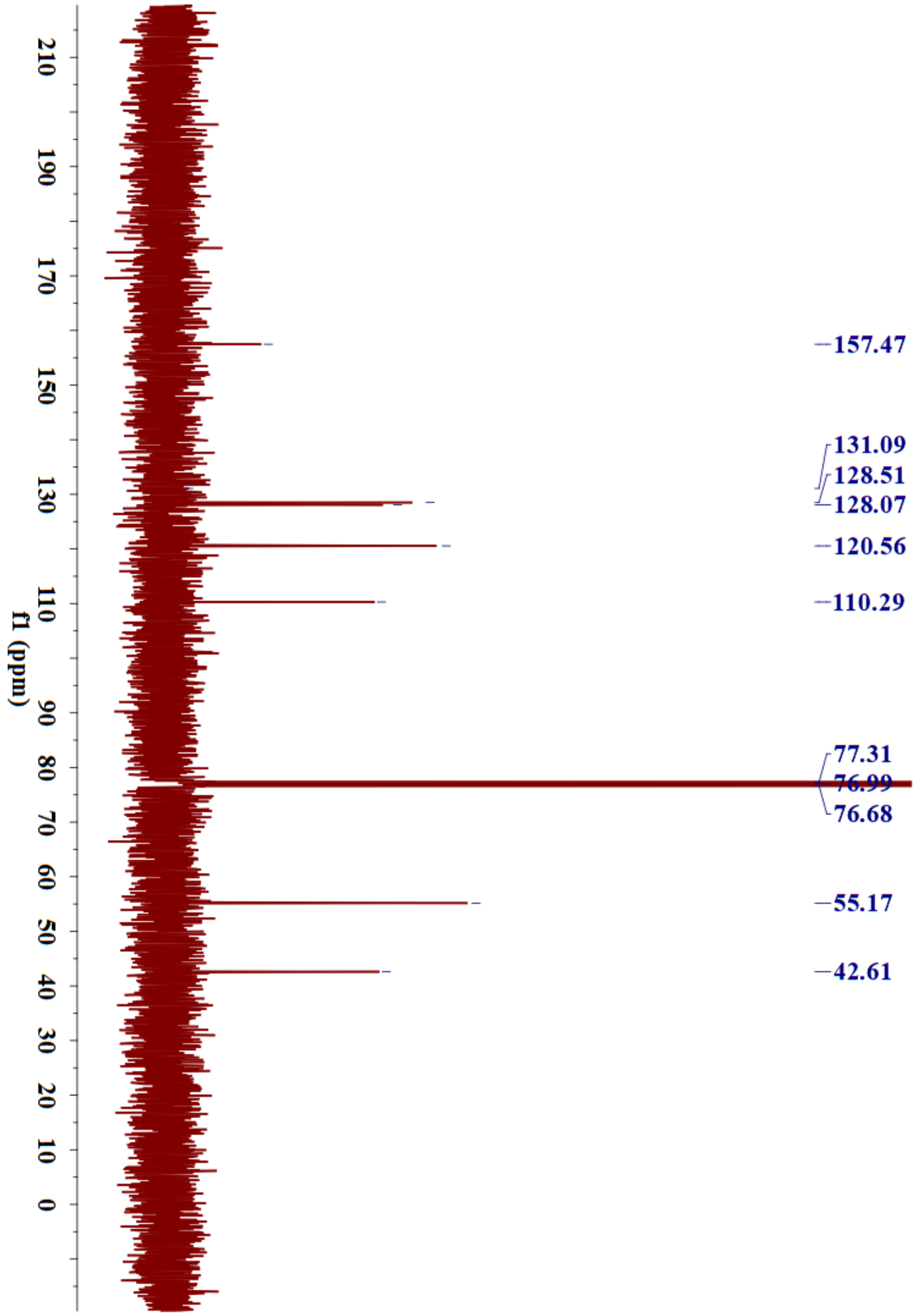

^{1}H NMR of 4-methoxybenzylamine (3e)

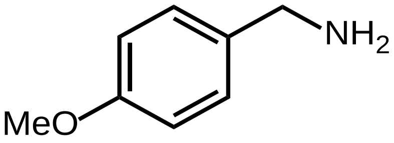

3e

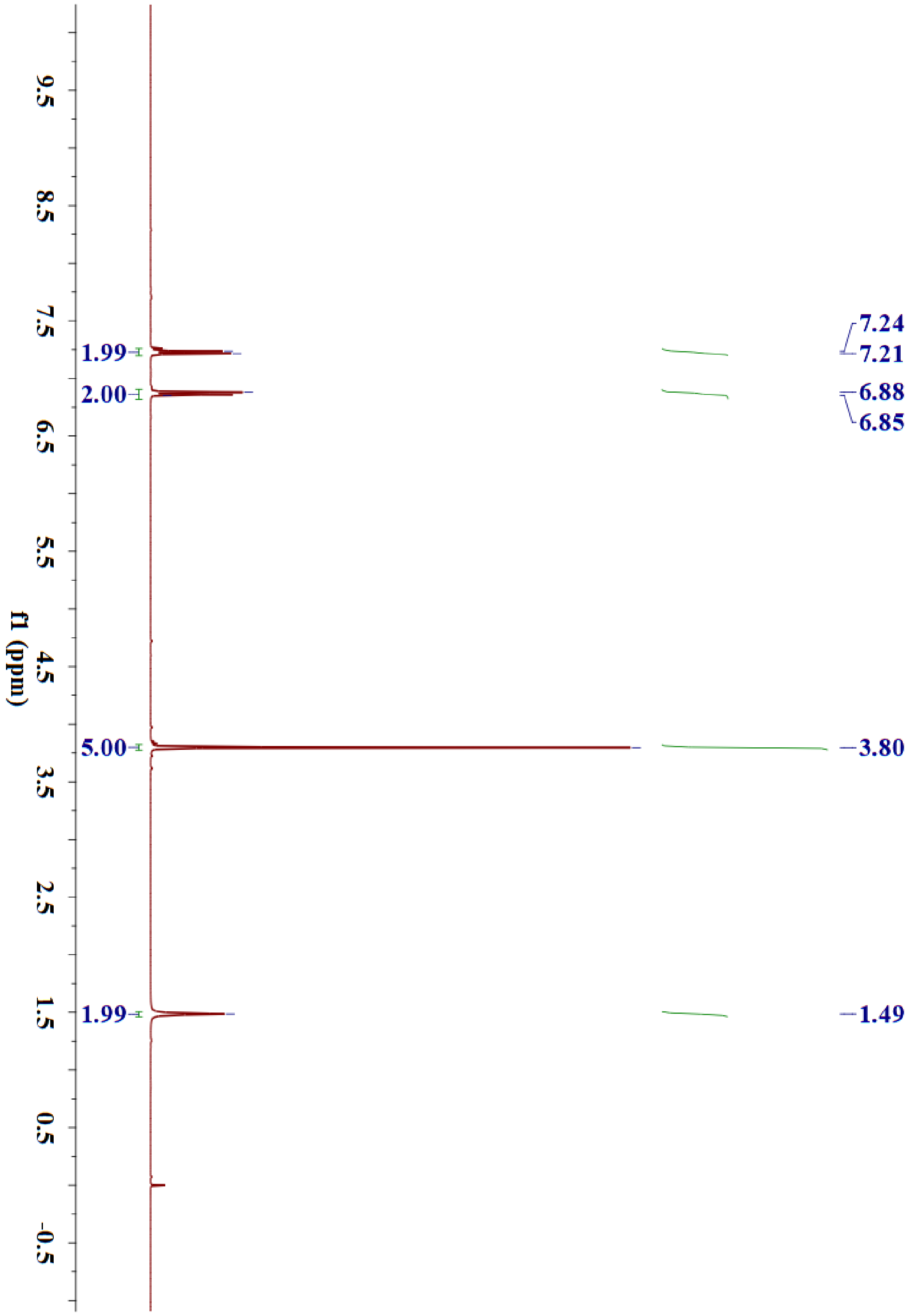

^{13}C NMR of 4-methoxybenzylamine (3e)

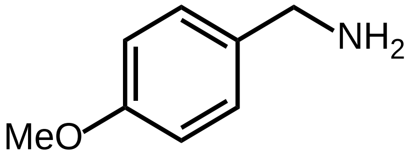

3e

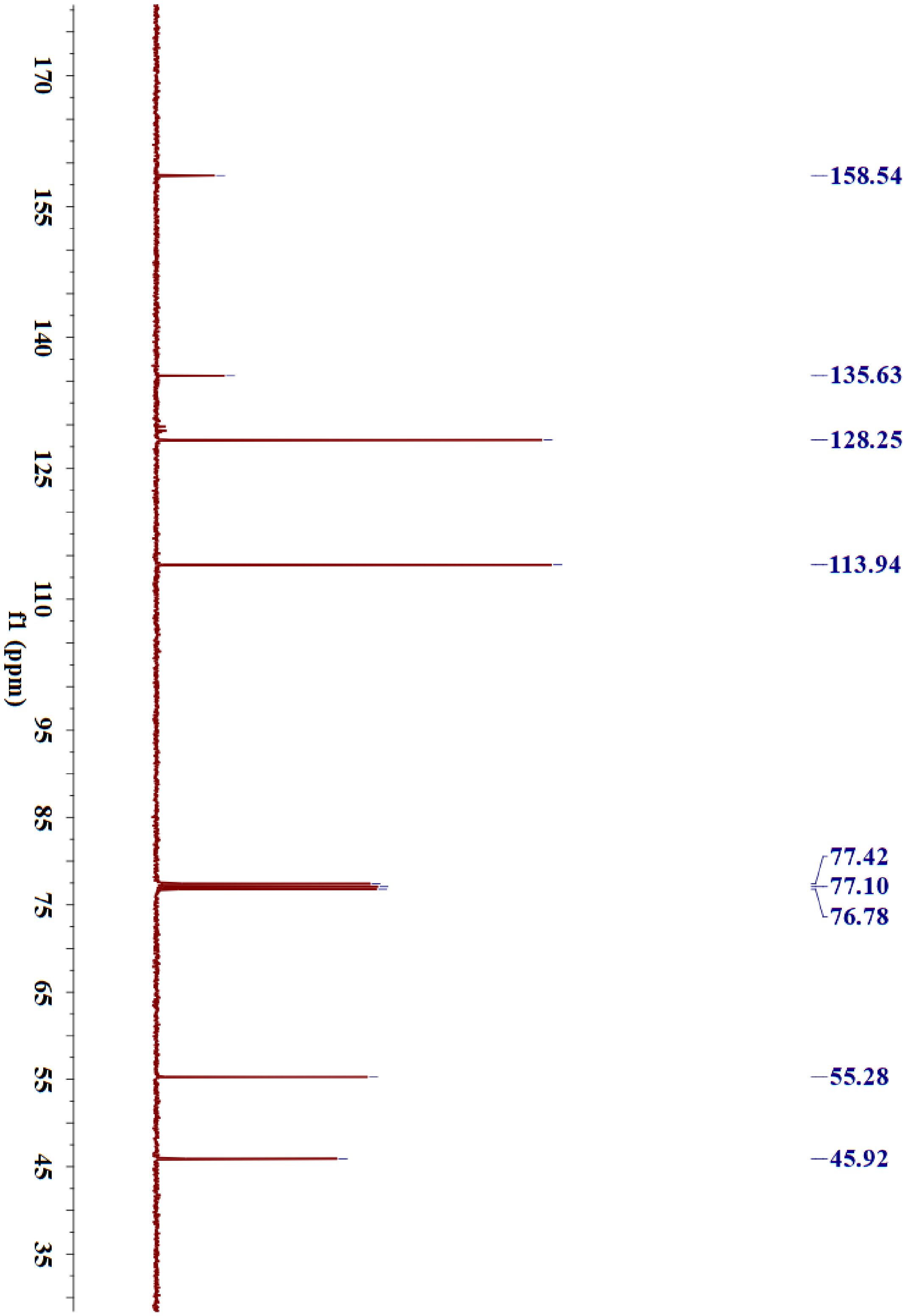

^{1}H NMR of ethyl 4-(aminomethyl)benzoate (3f)

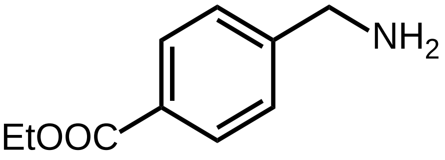

3f

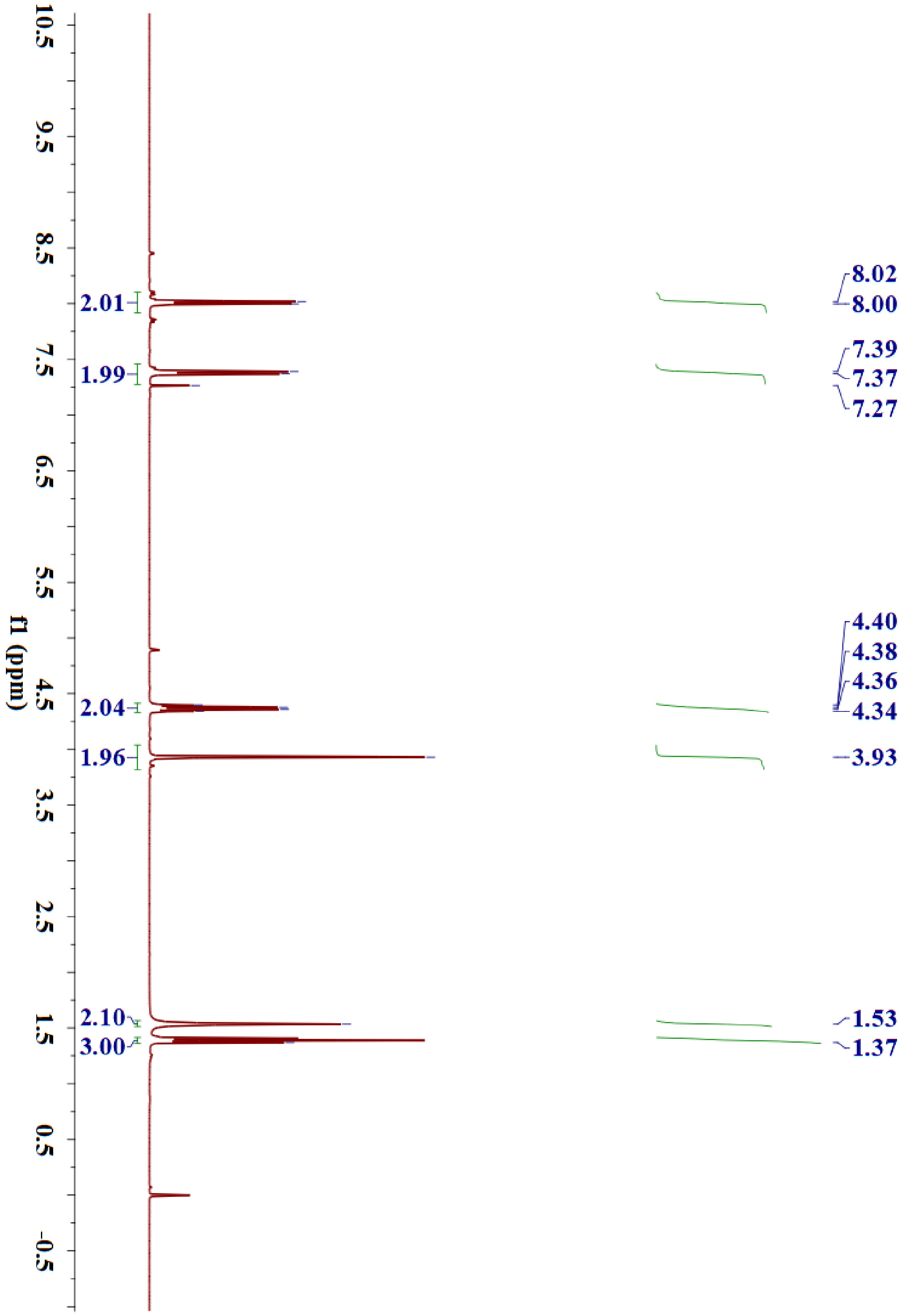

^{13}C NMR of ethyl 4-(aminomethyl)benzoate (3f)

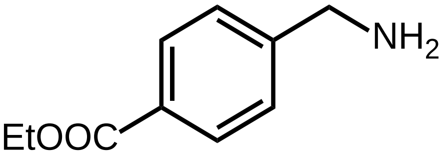

3f

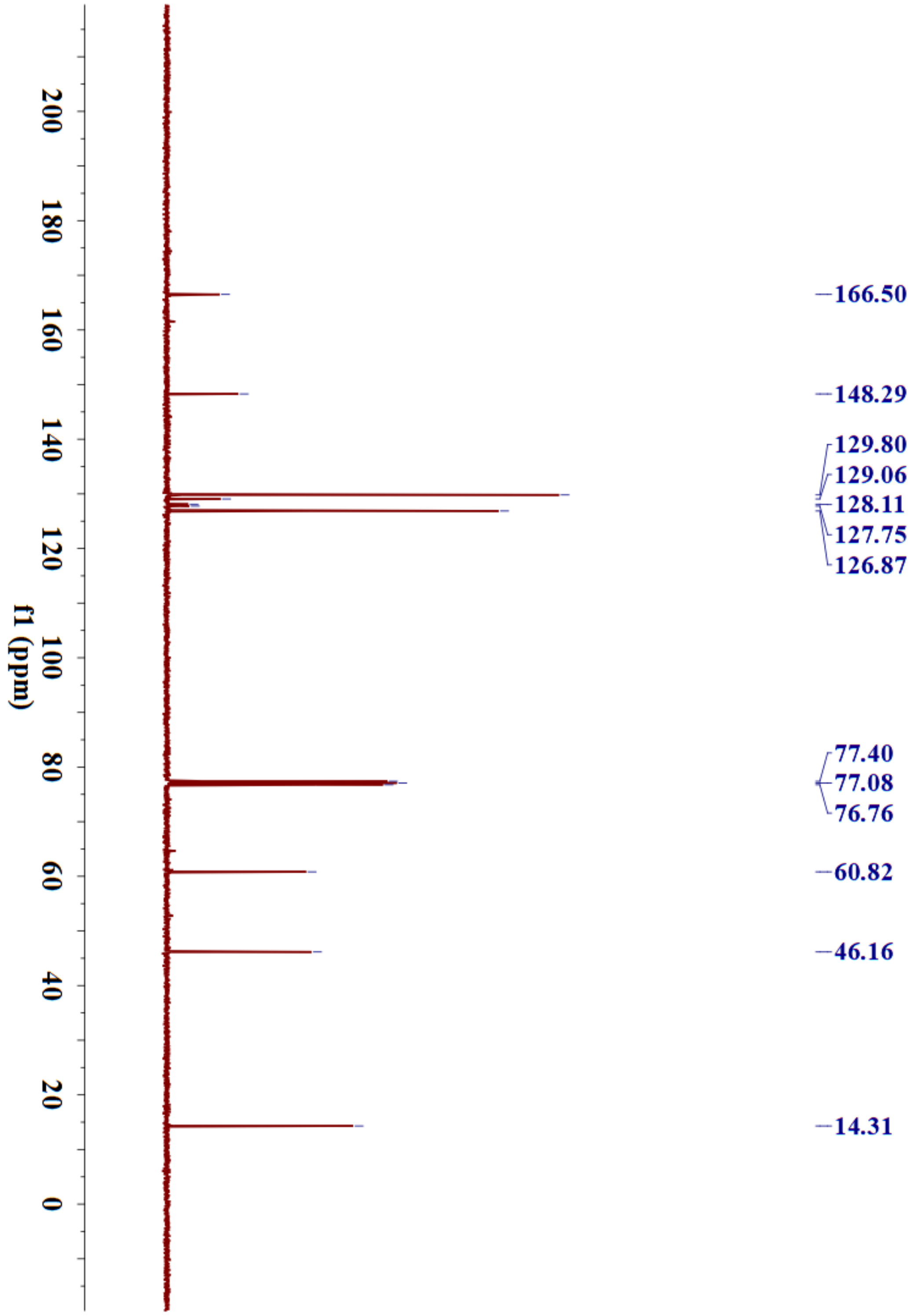

^{1}H NMR of 4-iodobenzylamine (3g)

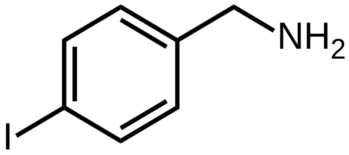

3g

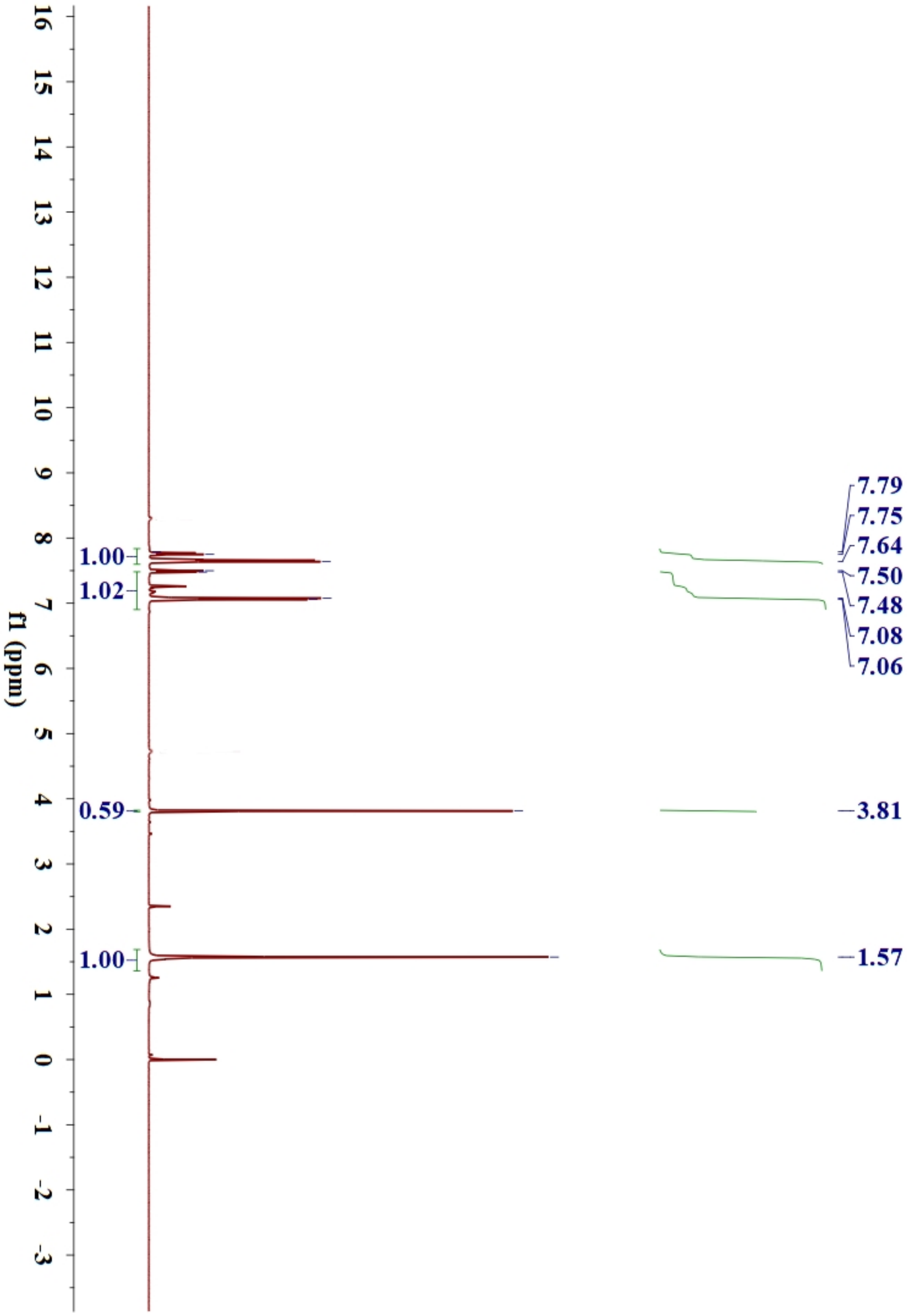

^{13}C NMR of 4-iodobenzylamine (3g)

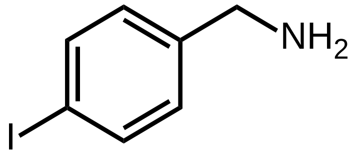

3g

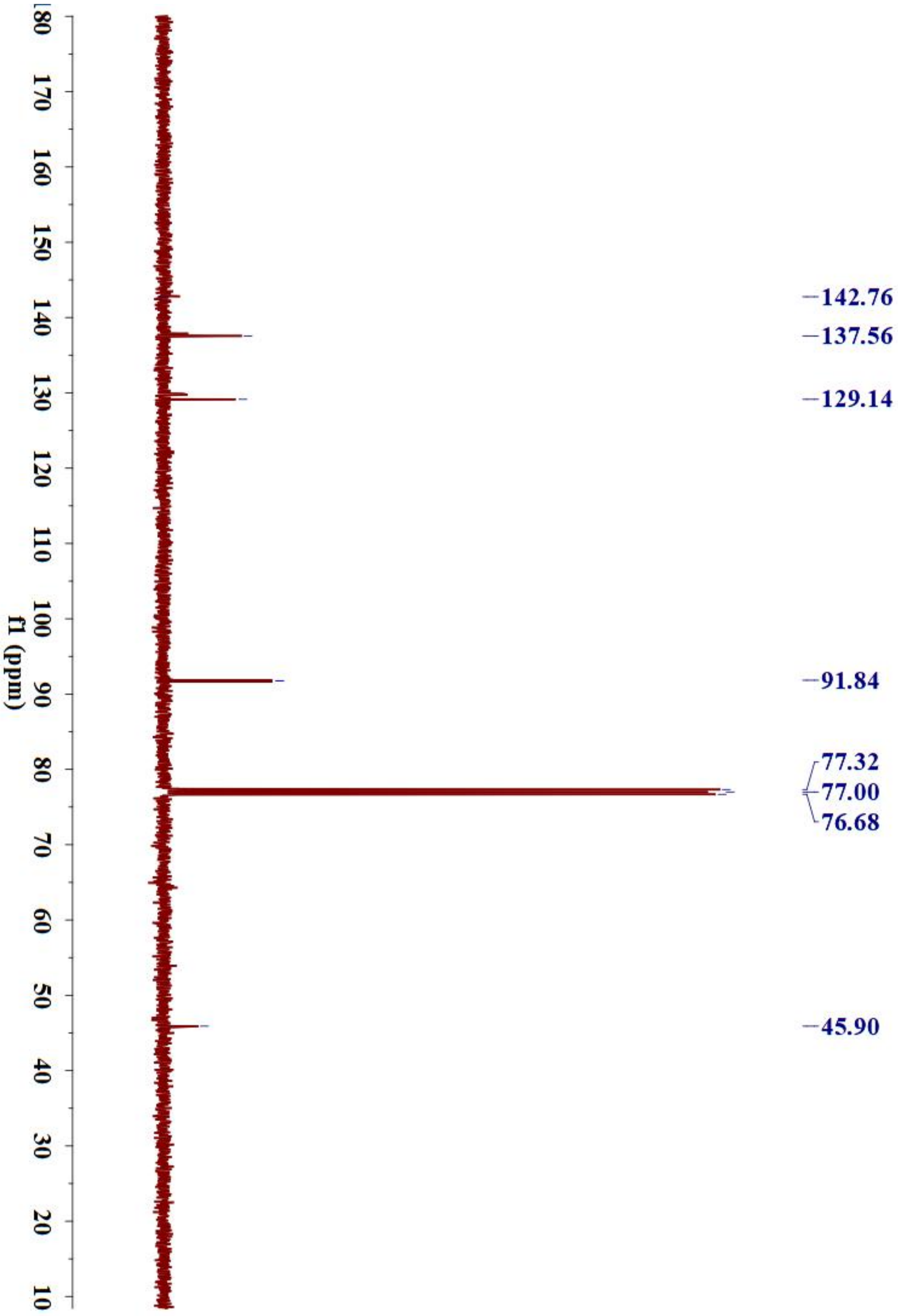

^{1}H NMR of 4-(aminomethyl)pyridine (**3h**)

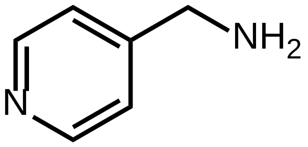

3h

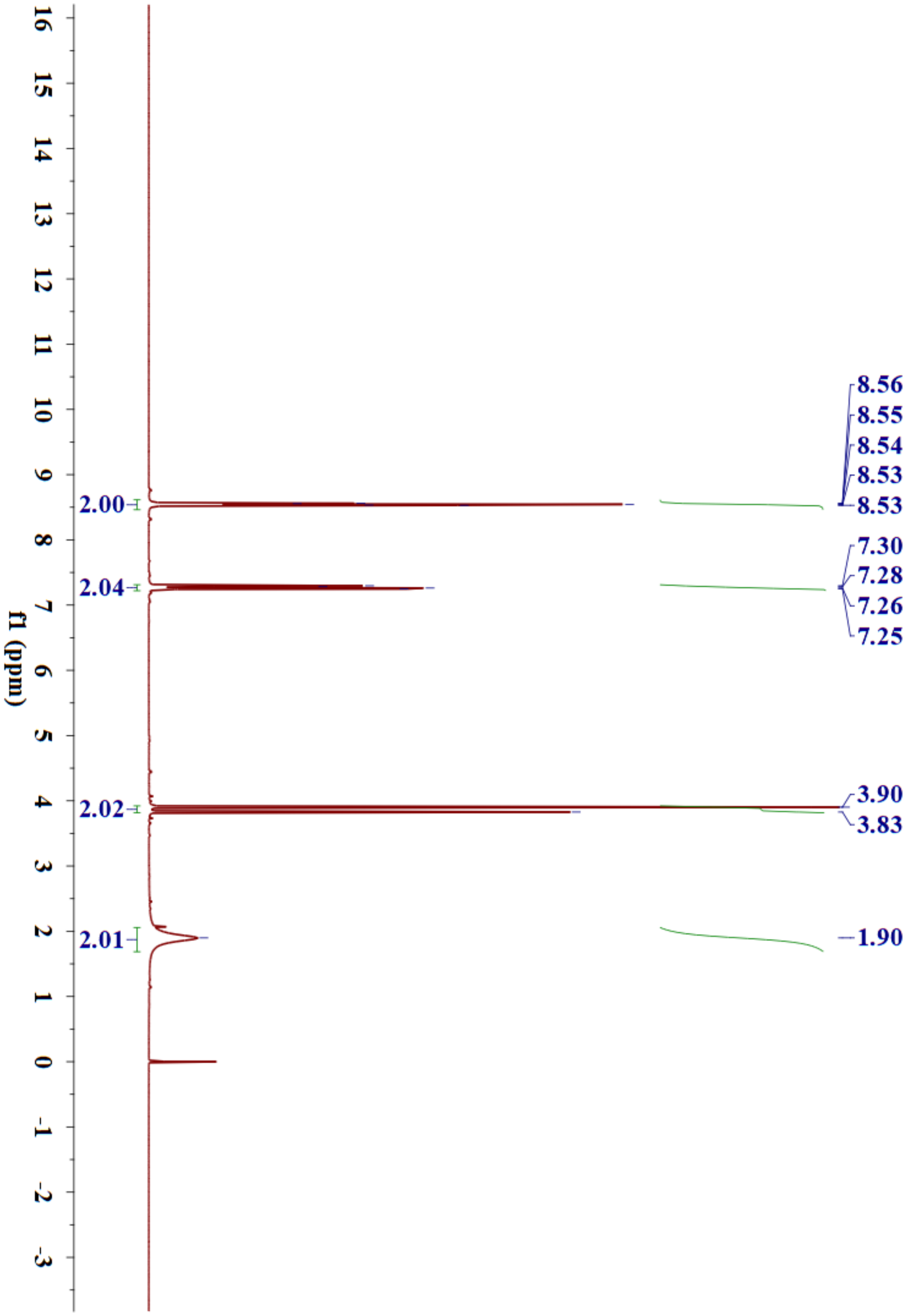

^{13}C NMR of 4-(aminomethyl)pyridine (**3h**)

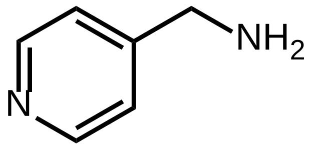

3h

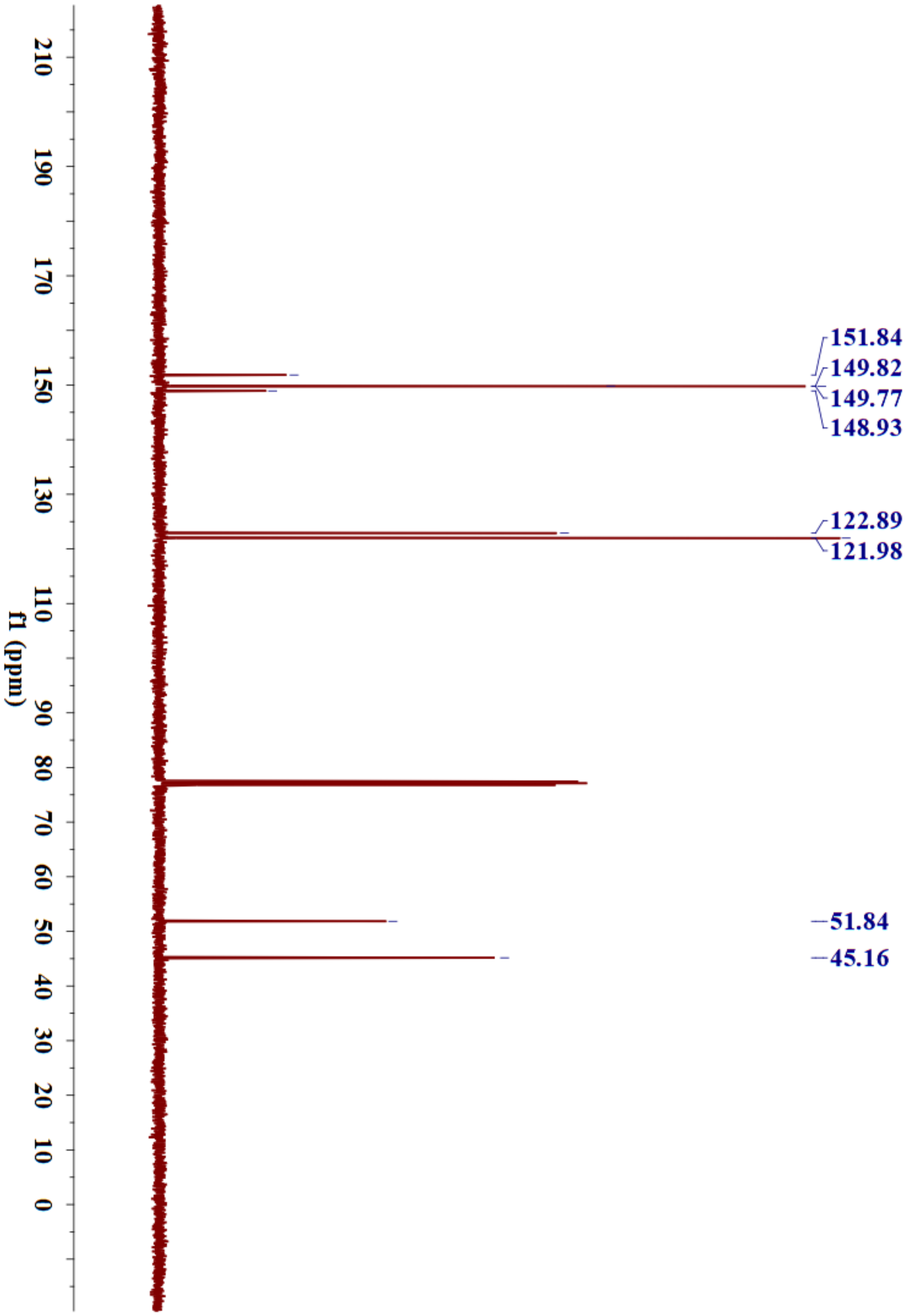

^{1}H NMR of Di(4-pyridylmethyl)amine (4h)

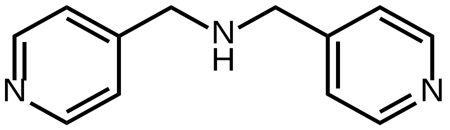

4h

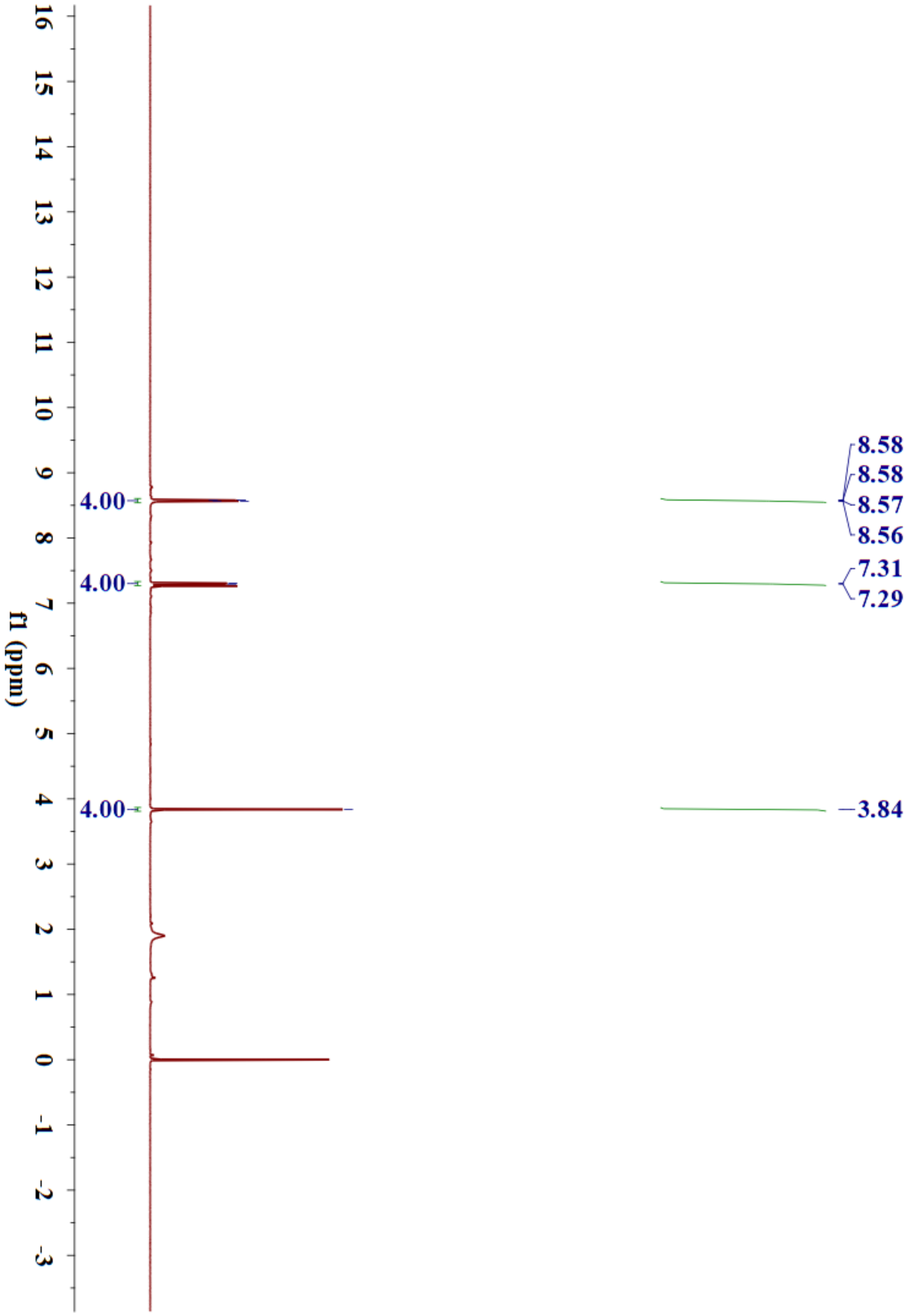

^{13}C NMR of Di(4-pyridylmethyl)amine (4h)

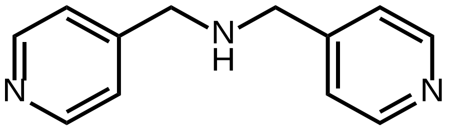

4h

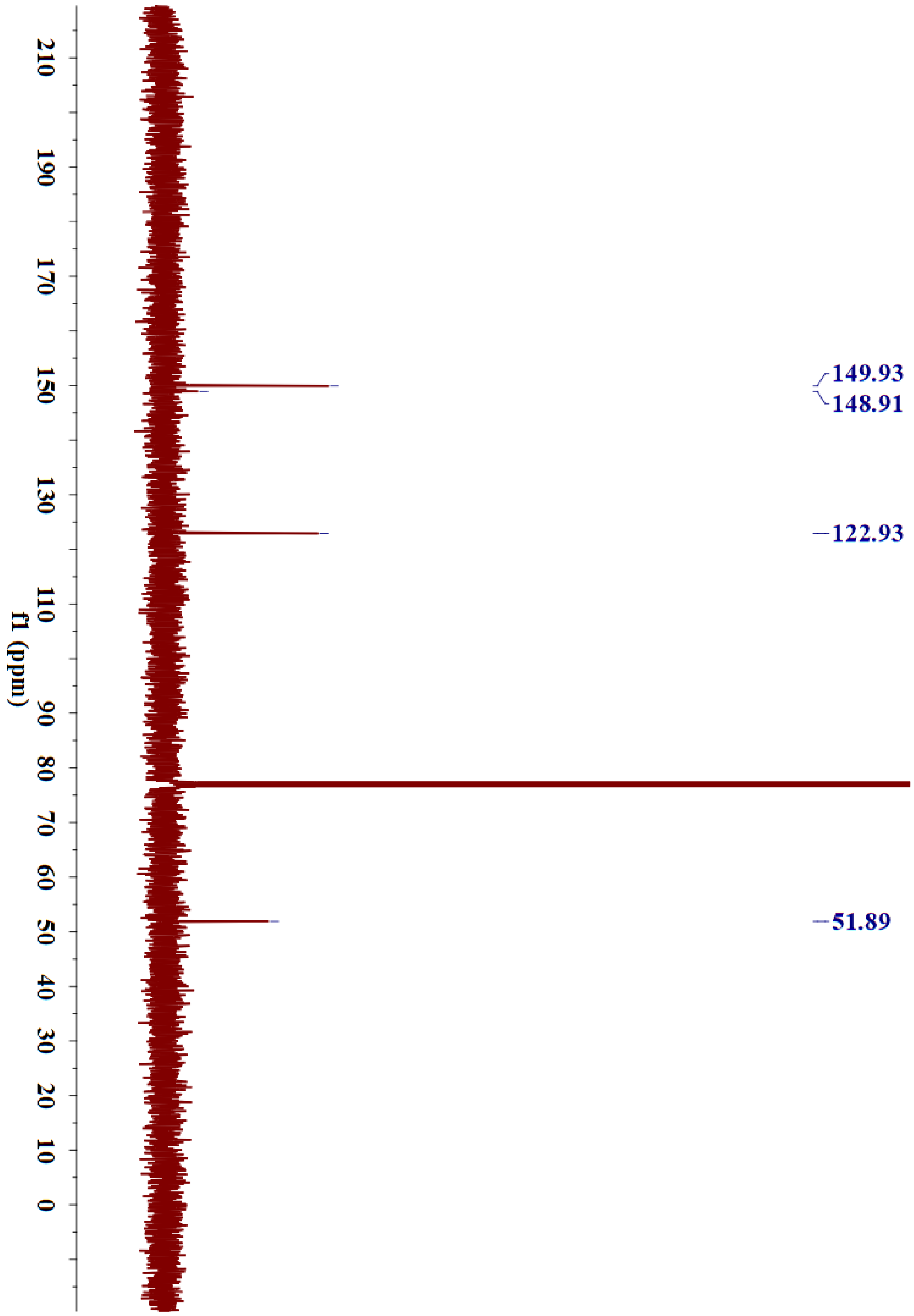

^{1}H NMR of phenethylamine (3i)

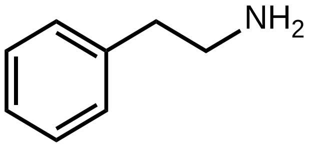

3i

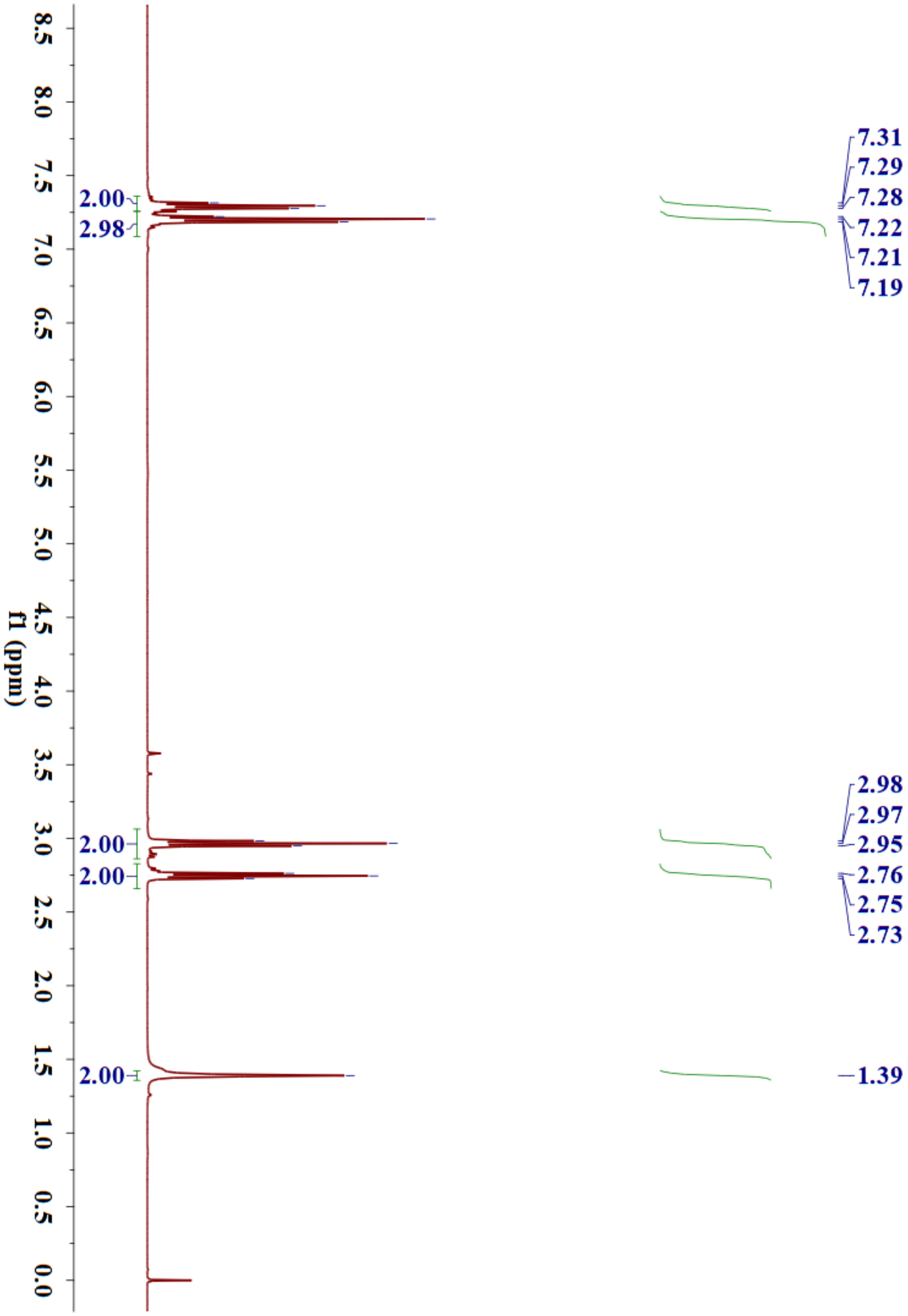

^{13}C NMR of phenethylamine (3i)

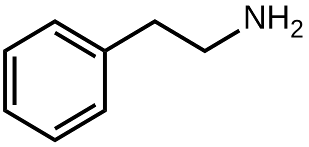

3i

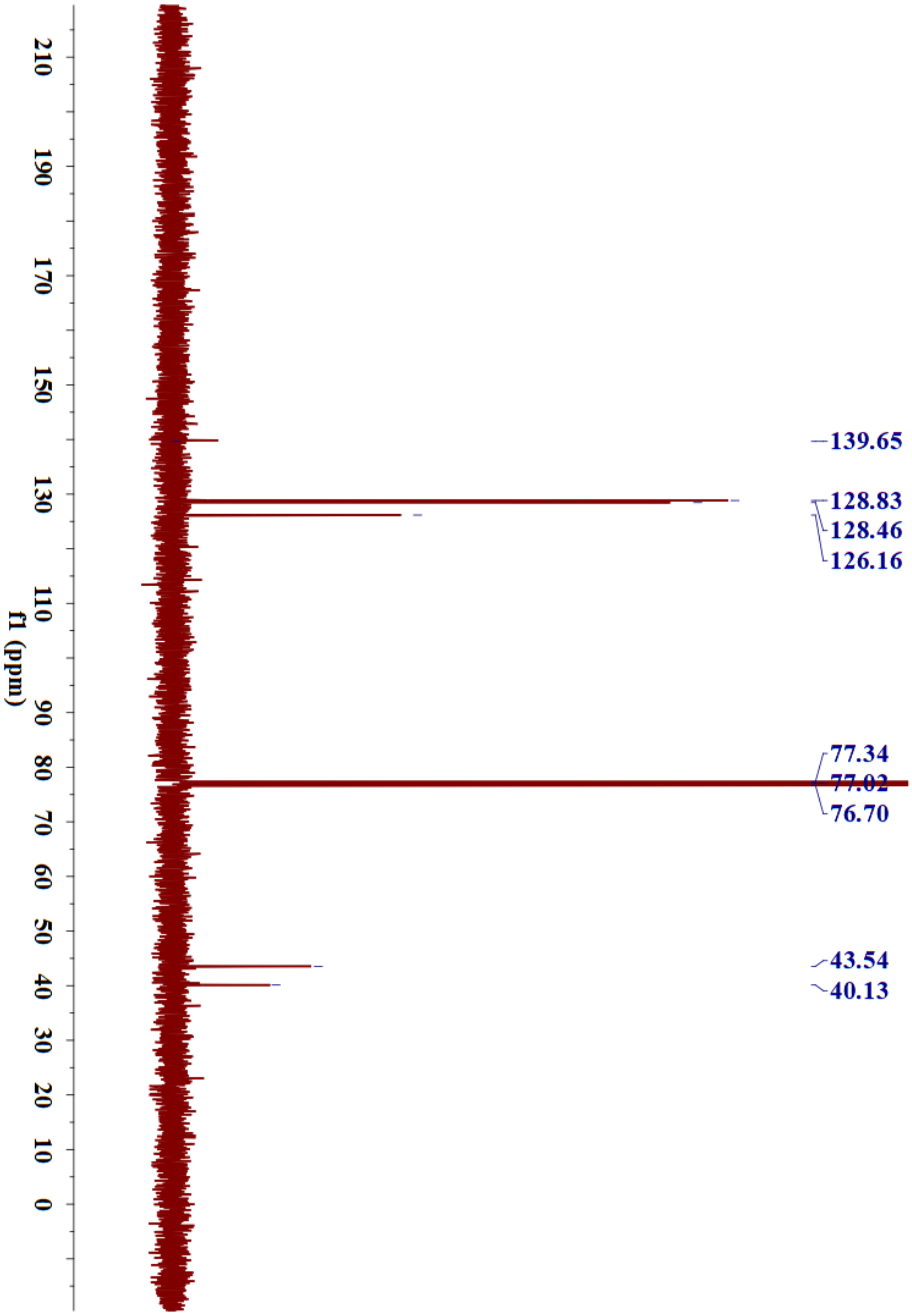

^{1}H NMR of n-octylamine (3j)

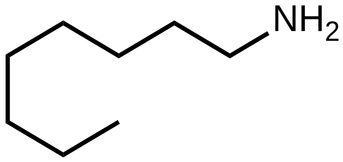

3j

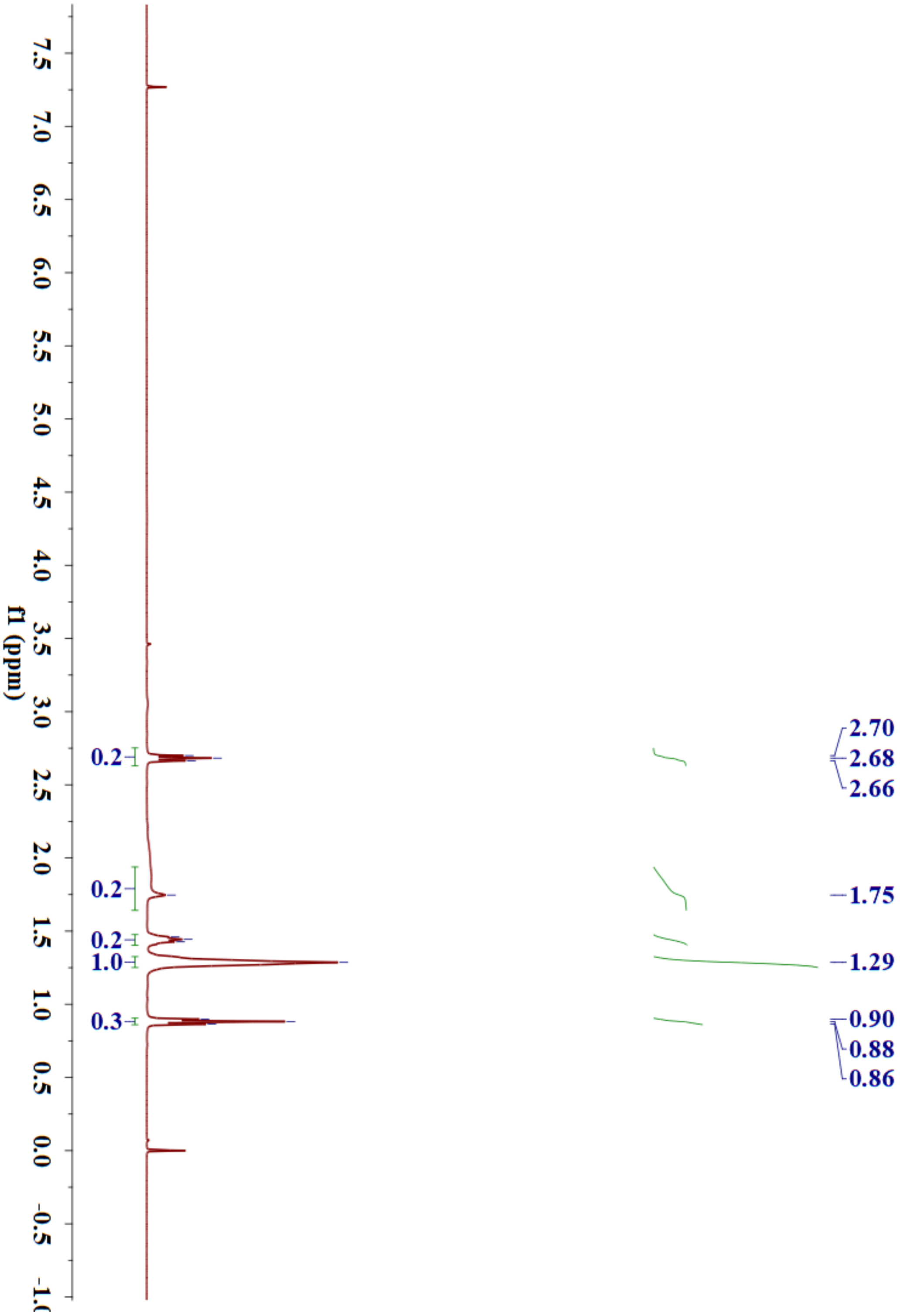

^{13}C NMR of n-octylamine (3j)

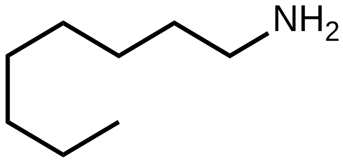

3j

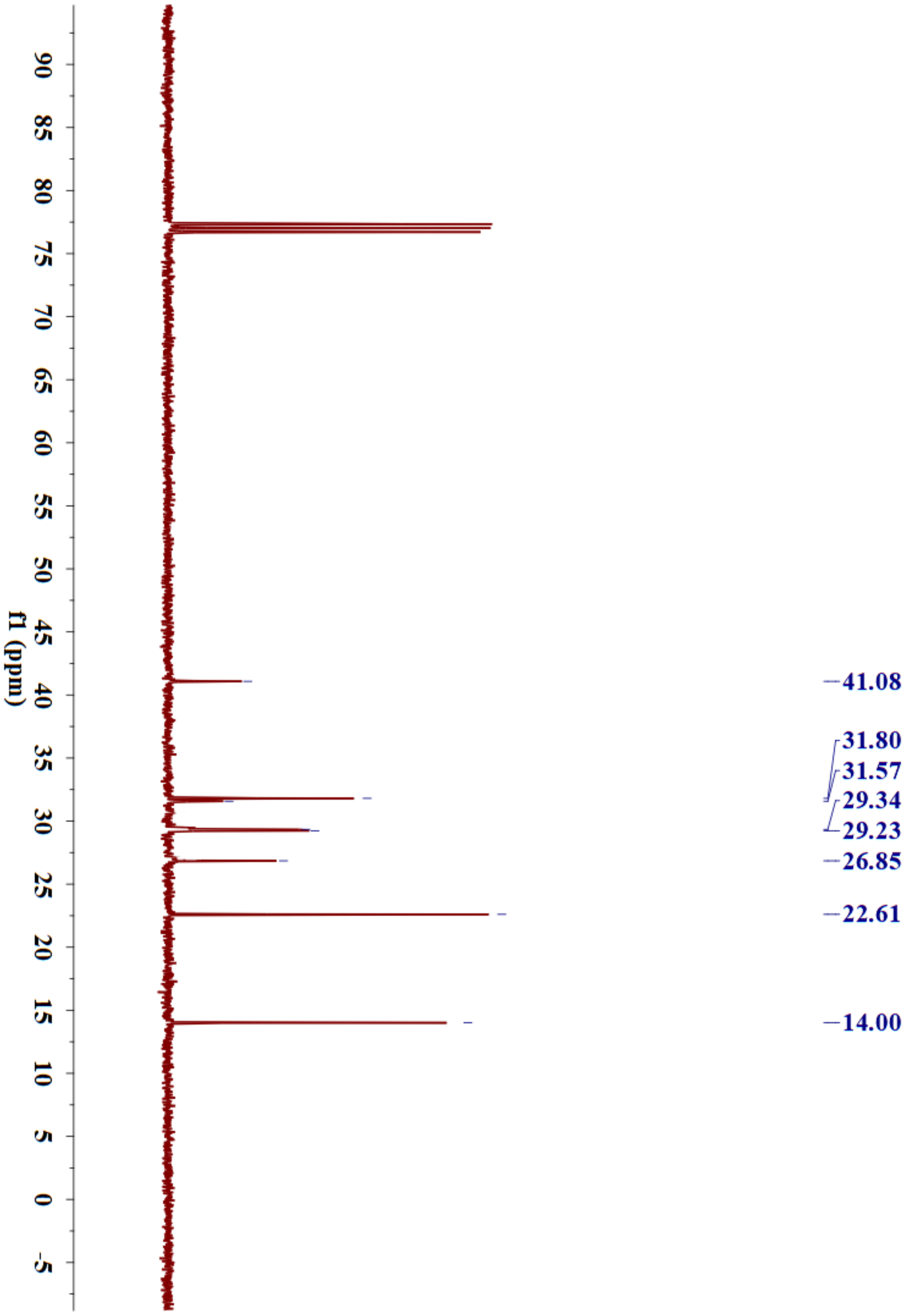

4. References

(1) Jiang, P.; Liu, Q.; Ge, C.; Cui, W.; Pu, Z.; Asiri, A. M.; Sun, X. CoP Nanostructures with Different Morphologies: Synthesis, Characterization and a Study of Their Electrocatalytic Performance toward the Hydrogen Evolution Reaction. *J. Mater. Chem. A* **2014**, *2*, 14634.

(2) Du, X.; Li, N.; Zhang, X. Controlled Synthesis of Co_3O_4@$NiMoO_4$ Core–Shell Nanorod Arrays for Efficient Water Splitting. *Dalton Trans.* **2018**, *47*, 12071–12074.

(3) Li, P.; Chen, R.; Lin, Y.; Li, W. General Approach to Construct Hierarchical-Structured Porous Co–Ni Bimetallic Oxides for Efficient Oxygen Evolution. *Inorg. Chem. Front.* **2020**, *7*, 2611–2620.

(4) Bhaskar, S.; Awin, E. W.; Kumar, K. C. H.; Lale, A.; Bernard, S.; Kumar, R. Design of Nanoscaled Heterojunctions in Precursor-Derived t-ZrO_2/SiOC(N) Nanocomposites: Transgressing the Boundaries of Catalytic Activity from UV to Visible Light. *Sci. Rep.* **2020**, *10*, 430.

(5) Liu, Y.; Yang, J. Hydrophobic Modification of ZrO_2-SiO_2 Xerogel and Its Adsorption Properties to Rhodamine B. *Gels* **2022**, *8*, 675.

(6) Redhead, P. A. Thermal Desorption of Gases. *VACUUM* **1962**, *12*, 203–211.

(7) Yang, K.; Chen, N.; Guo, X.; Zhang, R.; Sheng, X.; Ge, H.; Zhu, Z.; Yang, H.; Lü, H. Phase-Controlled Cobalt Catalyst Boosting Hydrogenation of 5-Hydroxymethylfurfural to 2,5-Dimethylfuran. *Molecules* **2023**, *28*, 4918.

(8) Meziane, L.; Salzemann, C.; Aubert, C.; Gérard, H.; Petit, C.; Petit, M. Hcp Cobalt Nanocrystals with High Magnetic Anisotropy Prepared by Easy One-Pot Synthesis. *Nanoscale* **2016**, *8*, 18640–18645.

(9) Guo, F.; Zheng, H.; Yang, Z.; Qian, Y. Synthesis of Cobalt Nanoparticles in Ethanol Hydrazine Alkaline System (EHAS) at Room Temperature. *Mater. Lett.* **2002**, *56*, 906–909.

(10) Sergiienko, R.; Shibata, E.; Zentaro, A.; Shindo, D.; Nakamura, T.; Qin, G. Formation and Characterization of Graphite-Encapsulated Cobalt Nanoparticles Synthesized by Electric Discharge in an Ultrasonic Cavitation Field of Liquid Ethanol. *Acta Mater.* **2007**, *55*, 3671–3680.

(11) Kato, K.; Deng, D.; Kita, Y.; Kamata, K.; Hara, M. Primary Amine Synthesis by Hydrogen-Involving Reactions over Heterogeneous Cobalt Catalysts. *Catal. Sci. Technol.* **2022**, *12*, 5425–5434.

(12) Sheng, M.; Yamaguchi, S.; Nakata, A.; Yamazoe, S.; Nakajima, K.; Yamasaki, J.; Mizugaki, T.; Mitsudome, T. Hydrotalcite-Supported Cobalt Phosphide Nanorods as a Highly Active and Reusable Heterogeneous Catalyst for Ammonia-Free Selective Hydrogenation of Nitriles to Primary Amines. *ACS Sustain. Chem. Eng.* **2021**, *9*, 11238–11246.

(13) Dai, H.; Guan, H. Switching the Selectivity of Cobalt-Catalyzed Hydrogenation of Nitriles. *ACS Catal.* **2018**, *8*, 9125–9130.

(14) Ji, P.; Manna, K.; Lin, Z.; Feng, X.; Urban, A.; Song, Y.; Lin, W. Single-Site Cobalt Catalysts at New $Zr_{12}(\mu_3\text{-}O)_8(\mu_3\text{-}OH)_8(\mu_2\text{-}OH)_6$ Metal–Organic Framework Nodes for Highly Active Hydrogenation of Nitroarenes, Nitriles, and Isocyanides. *J. Am. Chem. Soc.* **2017**, *139*, 7004–7011.

(15) Mitsudome, T.; Sheng, M.; Nakata, A.; Yamasaki, J.; Mizugaki, T.; Jitsukawa, K. A Cobalt Phosphide Catalyst for the Hydrogenation of Nitriles. *Chem. Sci.* **2020**, *11*, 6682–6689.

(16) Timelthaler, D.; Topf, C. Liquid-Phase Hydrogenation of Nitriles to Amines Facilitated by a Co(II)/Zn(0) Pair: A Ligand-Free Catalytic Protocol. *J. Org. Chem.* **2019**, *84*, 11604–11611.

(17) Ferraccioli, R.; Borovika, D.; Surkus, A.-E.; Kreyenschulte, C.; Topf, C.; Beller, M. Synthesis of Cobalt Nanoparticles by Pyrolysis of Vitamin B_{12}: A Non-Noble-Metal Catalyst for Efficient Hydrogenation of Nitriles. *Catal. Sci. Technol.* **2018**, *8*, 499–507.

(18) Murugesan, K.; Senthamarai, T.; Sohail, M.; Alshammari, A. S.; Pohl, M.-M.; Beller, M.; Jagadeesh, R. V. Cobalt-Based Nanoparticles Prepared from MOF–Carbon Templates as Efficient Hydrogenation Catalysts. *Chem. Sci.* **2018**, *9*, 8553–8560.

(19) Chen, F.; Topf, C.; Radnik, J.; Kreyenschulte, C.; Lund, H.; Schneider, M.; Surkus, A.-E.; He, L.; Junge, K.; Beller, M. Stable and Inert Cobalt Catalysts for Highly Selective and Practical Hydrogenation of C≡N and C═O Bonds. *J. Am. Chem. Soc.* **2016**, *138*, 8781–8788.

(20) Formenti, D.; Mocci, R.; Atia, H.; Dastgir, S.; Anwar, M.; Bachmann, S.; Scalone, M.; Junge, K.; Beller, M. A State-of-the-Art Heterogeneous Catalyst for Efficient and General Nitrile Hydrogenation. *Chem. – Eur. J.* **2020**, *26*, 15589–15595.

(21) Cai, W.; Yang, Y.; Ma, J.; Cao, J.; Ren, X.; Zhao, B. Transamination of Aromatic Aldehydes to Primary Arylmethylamines. *Org. Lett.* **2023**, *25*, 3876–3880.

(22) Wei, Z.; Li, H.; Wang, Y.; Liu, Q. A Tailored Versatile and Efficient NHC-Based NNC-Pincer Manganese Catalyst for Hydrogenation of Polar Unsaturated Compounds. *Angew. Chem. Int. Ed.* **2023**, *62*, e202301042.

(23) Mukherjee, A.; Srimani, D.; Chakraborty, S.; Ben-David, Y.; Milstein, D. Selective Hydrogenation of Nitriles to Primary Amines Catalyzed by a Cobalt Pincer Complex. *J. Am. Chem. Soc.* **2015**, *137*, 8888–8891.

(24) Gawel, J. M.; Shouksmith, A. E.; Raouf, Y. S.; Nawar, N.; Toutah, K.; Bukhari, S.; Manaswiyoungkul, P.; Olaoye, O. O.; Israelian, J.; Radu, T. B.; Cabral, A. D.; Sina, D.; Sedighi, A.; De Araujo, E. D.; Gunning, P. T. PTG-0861: A Novel $HDAC_6$-Selective Inhibitor as a Therapeutic Strategy in Acute Myeloid Leukaemia. *Eur. J. Med. Chem.* **2020**, *201*, 112411.

(25) Utsumi, T.; Noda, K.; Kawauchi, D.; Ueda, H.; Tokuyama, H. Nitrile Synthesis by Aerobic Oxidation of Primary Amines and *in Situ* Generated Imines from Aldehydes and Ammonium Salt with Grubbs Catalyst. *Adv. Synth. Catal.* **2020**, *362*, 3583–3588.

(26) Lu, Z.; Williams, T. J. A Dual Site Catalyst for Mild, Selective Nitrile Reduction. *Chem Commun* **2014**, *50*, 5391–5393.

(27) Shao, Z.; Fu, S.; Wei, M.; Zhou, S.; Liu, Q. Mild and Selective Cobalt-Catalyzed Chemodivergent Transfer Hydrogenation of Nitriles. *Angew. Chem. Int. Ed.* **2016**, *55*, 14653–14657.

(28) Szostak, M.; Sautier, B.; Spain, M.; Procter, D. J. Electron Transfer Reduction of Nitriles Using SmI_2–Et_3N–H_2O: Synthetic Utility and Mechanism. *Org. Lett.* **2014**, *16*, 1092–1095.
